# WNT-mediating TCF/LEF transcription factor gene expression in early human pluripotency and cell lineages differs from the rodent paradigm

**DOI:** 10.1242/jcs.264257

**Published:** 2025-09-26

**Authors:** Connor Ross, Paula A. Balestrini, Lawrence E. Bates, Takuya Azami, Taiye Adakole, Maxine Semple, Marika Salonna, Richard Gyuris, Jennifer Nichols, Norah E. Fogarty, Stefan Hoppler

**Affiliations:** ^1^ Institute of Medical Sciences, Foresterhill Health Campus, University of Aberdeen, Aberdeen AB25 2ZD, UK; ^2^ Centre for Gene Therapy and Regenerative Medicine, King's College London, Tower Wing, Guy's Maze Pond, London SE1 9RT, UK; ^3^ MRC Human Genetics Unit, Institute of Genetics and Cancer, The University of Edinburgh, Western General Hospital, Crewe Road, Edinburgh EH4 2XU, UK; ^4^ Guy's Assisted Conception Unit, Division of Women's Health, School of Medicine, King's College London, London SE1 9RT, UK

**Keywords:** Human development, Naïve pluripotency, WNT/β-catenin signalling, TCF/LEF transcription factors, Extra-embryonic cell lineages, Early embryonic cell lineages

## Abstract

Embryonic stem (ES) cell research has uncovered different requirements for WNT/β-catenin signalling in human naïve pluripotent cells compared to the mouse paradigm. It is therefore important to study WNT/β-catenin signalling directly in models that recapitulate early human development. Since TCF/LEF transcription factors mediate regulation of target genes downstream of WNT/β-catenin signalling, we examined the regulation, expression and protein localisation of the four TCF/LEF genes by analysing *in vitro* ‘snapshots’ of human development, leveraging naïve and primed pluripotent cells, blastoids and preimplantation blastocysts. Strikingly, we comprehensively confirm clear differences between mouse and human pluripotent stem cells, suggesting their differential requirements for WNT signalling reflects a pluripotent state-dependent manner. Human naïve ES cells express considerably lower levels of TCF7L1, unlike their mouse counterparts. TCF7L2 is robustly expressed in the trophectoderm derived from naïve ES cells, in blastoids and human preimplantation blastocysts. In primed pluripotent stem cells, active WNT/β-catenin signalling induces the expression of both *TCF7* and *LEF1*, concomitant with hallmark gastrulation markers. The expression of human TCF/LEF genes indicates a differential requirement for WNT/β-catenin signalling throughout early human embryo development that warrants further investigation.

## Teaser

**Summary:** Analysis of TCF/LEF factors unearths differences between mouse and human pluripotent stem cells, indicating differential requirements between species for WNT/β-catenin signalling in early embryo development.

## INTRODUCTION

WNT signalling is a widely conserved biochemical cell-to-cell signalling mechanism, which mediates diverse biological functions including during early embryonic development and later tissue stem cell-mediated regeneration and disease (reviewed by Hoppler and Moon, 2014; Nusse and Clevers, 2017). WNT/β-catenin signal transduction stabilises and drives free cytoplasmic β-catenin protein into the nucleus to regulate transcription of context-specific WNT target genes in association with sequence-specific DNA-binding factors, primarily with the T Cell Factor (TCF) and Lymphoid Enhancer Factor (LEF) family (collectively referred to as TCF/LEF; Cadigan and Waterman, 2012; Hoppler and Waterman, 2014). These function as bimodal transcription factors (i.e. repressing target gene expression without nuclear β-catenin and activating target gene expression with nuclear β-catenin; Ramakrishnan and Cadigan, 2017). In vertebrates, there are four TCF/LEF genes – *TCF7* (formerly *TCF1*), *TCF7L1* (formerly *TCF3*), *TCF7L2* (formerly *TCF4*) and *LEF1* (Torres-Aguila et al., 2022) – which mediate different biological functions in a cell- and context-dependent manner that is not yet understood (reviewed by Hoppler and Waterman, 2014).

Stem cells isolated from the early embryo provide accessible experimental models for studying early embryonic development in cell culture, especially in the context of early human development. Stable stem cell cultures originally isolated from mouse embryos (Evans and Kaufman, 1981; Martin, 1981) and those first derived from human blastocysts differ in several aspects (Thomson et al., 1998), and they have subsequently been recognised to represent different states of pluripotency – naïve and primed, respectively (Nichols and Smith, 2009). Human pluripotent stem cells (hPSCs) can be cultured in conditions supportive of a naïve state (Bayerl et al., 2021; Bredenkamp et al., 2019; Guo et al., 2016, 2017; Khan et al., 2021; Takashima et al., 2014; Theunissen et al., 2014) but, unlike mouse naïve embryonic stem (ES) cells, they have unrestricted potential to differentiate into extra-embryonic lineages (Dattani et al., 2024; Guo et al., 2021; Io et al., 2021; Linneberg-Agerholm et al., 2019).

Some of these differences in the naïve state between human and mouse models involves WNT/β-catenin signalling. For instance, WNT pathway activation via CHIR99021 (as part of the 2i+LIF culture conditions; CHIR99021 is also referred to herein as CHIR) directly supports the long-term propagation of mouse naïve ES cells by boosting expression of key target genes such as *Esrrb*, *Klf2* and *Nanog* through relieving the inhibitory activity of Tcf7l1 (Cole et al., 2008; Martello et al., 2012; Wray et al., 2011; Yi et al., 2011). Interestingly, withdrawal of CHIR from 2i+LIF or depletion of functional β-catenin upregulates postimplantation genes (Tao et al., 2020). In contrast, human naïve ES cells cannot be propagated in conventional mouse 2i+LIF conditions and, rather, benefit from titrated levels of CHIR (Takashima et al., 2014). Further culture refinements through exchanging CHIR for XAV939, a potent tankyrase inhibitor that blocks WNT signalling (Huang et al., 2009) and Hippo signalling (Pillay et al., 2022; Wang et al., 2015), improves naïve hPSC expansion and homogeneity (Guo et al., 2017; Khan et al., 2021) whilst preventing spontaneous trophectoderm induction (Dattani et al., 2022). Finally, naïve hPSCs lacking β-catenin exhibit no reduction in key pluripotency marker or SUSD2 expression (Dattani et al., 2022).

Despite these clear species-specific differences in WNT signalling function, our understanding of the pathway in early human development is lacking. Here, we define the expression and localisation patterns of all four TCF/LEF transcription factors during directed differentiation from naïve and primed hPSCs into extra-embryonic and early embryonic cell lineages, respectively. This descriptive analysis will provide a benchmark for studying WNT signalling in human stem cell models and reveals how these patterns might deviate from the mouse model-derived paradigm. Our findings confirm clear differences with mouse and provide a solid conceptional platform for future investigations into the functional role of WNT signalling in early human embryogenesis.

## RESULTS

### Comparison of TCF/LEF expression between mouse and human in naïve and primed pluripotent stem cells

**Fig. 1.**
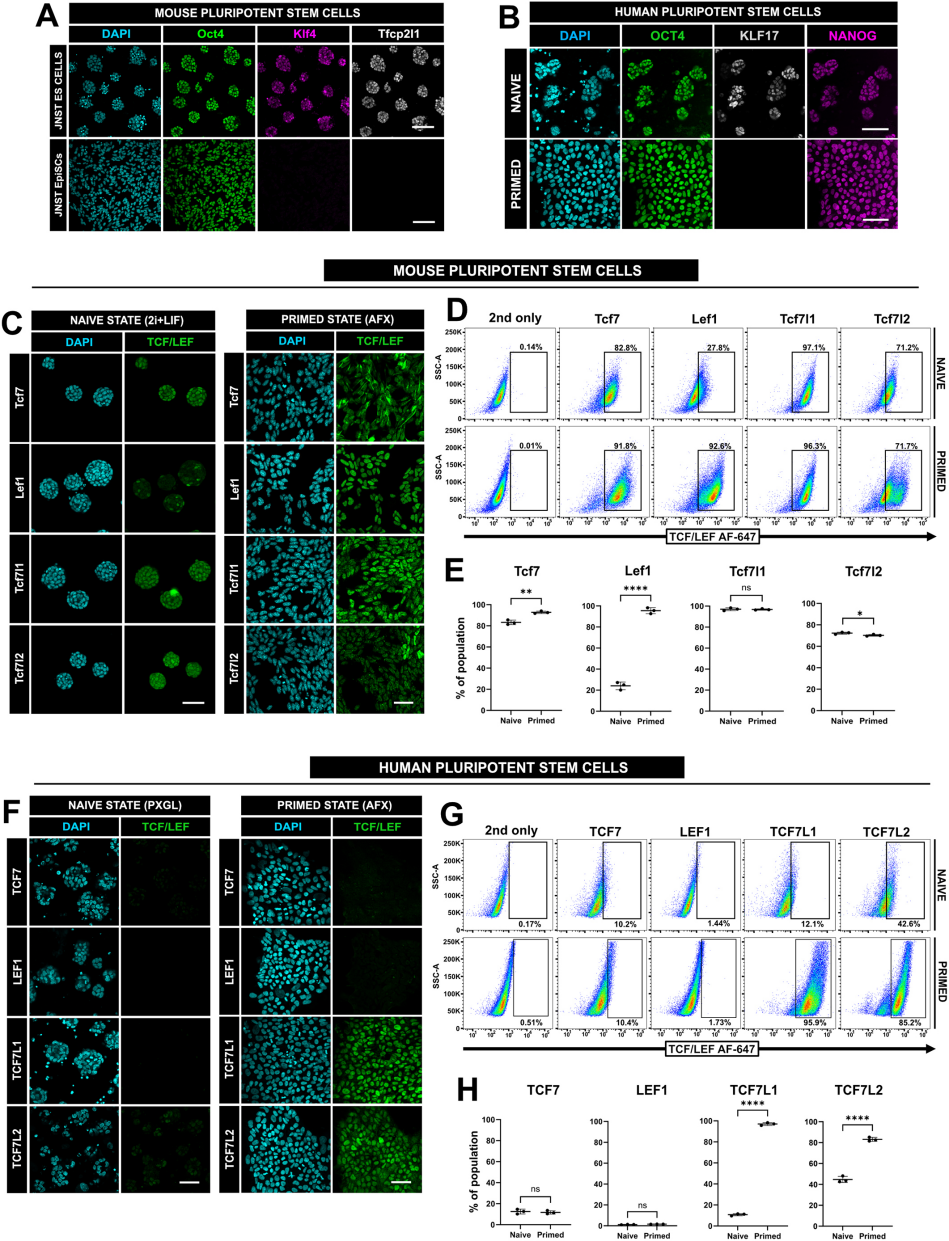
**Difference in TCF/LEF expression between human and mouse in both naïve and primed ES cells.** (A) Immunofluorescence of JNST #32 mESCs cultured in either 2i+LIF (naïve, top) or AFX (primed, bottom) for 48 h. Cells are labelled for Oct4 (green), Klf4 (magenta) and Tfcp2l1 (grey), and with DAPI (blue). Scale bars: 100 μm. (B) Immunofluorescence of HNES1 naïve ES cells labelled for OCT4 (green), KLF17 (grey) and NANOG (magenta), and with DAPI (blue), after 72 h of culture in PXGL (naïve) or AFX/StemFlex medium (primed). Scale bars: 100 μm. (C) Immunofluorescence of JNST mESCs cultured in 2i+LIF (naïve) or AFX (primed) conditions after 72 h. Cells are labelled for Tcf7, Lef1, Tcf7l1 and Tcf7l2, and with DAPI. Scale bars: 100 μm. (D) Distribution plots of mouse naïve and primed cells for each TCF/LEF factor as indicated. *N*=3. Percentages of cells within the marked TCF/LEF-positive gates are shown. 2nd only, secondary-antibody-only control. (E) Quantification of gated cell populations from flow cytometry data. One-tailed Student's paired *t*-test (*P<0.05; **P<0.01; ****P<0.0001; ns, not significant). Mean±s.d., *N*=3. (F) Immunofluorescence of HNES1 cells cultured in either PXGL (naïve) or StemFlex (primed) medium, and labelled for each of the TCF/LEF factors and with DAPI, as indicated. *N*=3. Scale bars: 100 μm. (G) Distribution plots of human naïve and primed cells for each TCF/LEF factor. *N*=3. Percentages of cells within the marked TCF/LEF-positive gates are shown. 2nd only, secondary-antibody-only control. (H) Quantification of gated cell populations for each TCF/LEF factor in human naïve and primed cells using flow cytometry. One-tailed Student's paired *t*-test (****P<0.0001; ns, not significant). Mean±s.d., *N*=3. Images in A–C, F are representative of five independent fields of view.

The expression of mutually exclusive naïve and primed markers can be used to characterise different pluripotent states. Mouse ES cells (mESCs, naïve state) show expression of Klf4 and Tfcp2l1, which is lost when cells are cultured in primed conditions (AFX: culture with Activin A, Fgf2 and XAV939), but both populations maintain Oct4 expression (Fig. 1A). Human pluripotent cells in both naïve and primed states express NANOG and OCT4, whereas KLF17 is restricted to the naïve state (Fig. 1B) (Blakeley et al., 2015; Stirparo et al., 2018). First, we evaluated the expression of all four TCF/LEF factors in mouse naïve ESCs and primed epiblast stem cells (mEpiSCs).

Immunofluorescence revealed expression of Tcf7 and Tcf7l1 in both naïve and primed states (Fig. 1C), supported by flow cytometry (Fig. 1D). We observed a significant increase in the number of Tcf7-positive cells between mESCs and mEpiSCs (Fig. 1D,E), whereas the incidence ofTcf7l1-positive cells remained unchanged (Fig. 1D,E). Tcf7l2 in mESCs was homogeneous, whereas in mEpiSCs, we detected two separate populations, which was also confirmed with flow cytometry (Fig. 1D). Interestingly, immunofluorescence of Lef1 in mESCs revealed a clearly heterogenous pattern, different from mEpiSCs (Fig. 1C; Fig. S1B). Flow cytometry faithfully captured these populations (Fig. 1D), confirming an increase in the number of Lef1-positive cells in mEpiSC conditions (Fig. 1E). Our observations align with previously reported expression profiles of the TCF/LEF genes in mESCs (Kelly et al., 2011; Moreira et al., 2017; Wallmen et al., 2012) and go beyond these previous reports by identifying heterogenous Lef1 expression.

Expression of TCF/LEF factors in naïve and primed hPSCs differs from that observed in mouse (Fig. 1F). Immunofluorescence of naïve hPSCs cultured in PXGL (PD0325901, XAV939, Gö6983 and LIF) revealed very low TCF7 levels (Fig. 1F). TCF7L2 signal was detected using flow cytometry, whereas TCF7L1 was largely absent except for a very small and low-expressing population, and LEF1 was undetectable (Fig. 1G). We then compared TCF/LEF expression in naïve hPSCs cultured in t2iLiGö (Takashima et al., 2014), the predecessor of PXGL that contains CHIR99021 (WNT activator) in lieu of XAV939 (WNT inhibitor) (Guo et al., 2017), to investigate whether modulating WNT activity can alter TCF/LEF expression. Naïve hPSCs were adapted over five passages to t2iLiGö (Fig. S1A) and retained their characteristic dome-shaped morphology (Fig. S1B). Immunofluorescence of PXGL and t2iLiGö cells confirmed co-expression of the naïve markers KLF17 and SUSD2 (Fig. S1C). Quantitative PCR (qPCR) analysis showed that expression of naïve and core pluripotent genes remained largely unchanged and *AXIN2* (a transcriptional readout for WNT/β-catenin signalling) was slightly upregulated in t2iLiGö (Fig. S1D). Further qPCR analysis revealed no distinct changes in *TCF7L1*, and *LEF1* was not detected at the mRNA level (Fig. S1E). Moreover, *TCF7* was slightly increased in t2iLiGö cells, compared to PXGL, with a small decrease in *TCF7L2* (Fig. S1E). Immunofluorescence of TCF/LEF proteins was largely comparable in PXGL and t2iLiGö cells, with the exception of lower TCF7L2 signal (Fig. S1F) and occasional LEF1-positive NANOG-negative cells present in t2iLiGö cells but not PXGL cultures (Fig. S1G). Flow cytometric analysis corroborated our qPCR data, demonstrating a reduction in TCF7L2-positive cells between PXGL (∼45%) and t2iLiGö (∼23%) (Fig. S1H,I).

Immunofluorescence of primed hPSCs revealed consistent nuclear localisation of TCF7L1 and TCF7L2 (Fig. 1F). At the population level, we detected both TCF7L1, corroborating previous observations (Sierra et al., 2018), and TCF7L2. In stark contrast to mouse primed cells, immunofluorescence of primed hPSCs demonstrated very low TCF7 expression and undetectable LEF1 expression (Fig. 1F), supported by flow cytometry (Fig. 1G,H).

Taken together, these data indicate distinct expression patterns for the TCF/LEF factors between mouse and human pluripotent stem cells, which may account for their opposing requirements for WNT signalling in order to maintain a naïve pluripotent state (Guo et al., 2017; Takashima et al., 2014; Ying et al., 2008). These differences between human and the mouse paradigm in the expression of TCF/LEF factors in both the naïve and primed states of pluripotency motivate and encourage a more detailed analysis of human TCF/LEF expression in differentiation of extra-embryonic and embryonic lineages.

### TCF/LEF expression in hypoblast cells

**Fig. 2.**
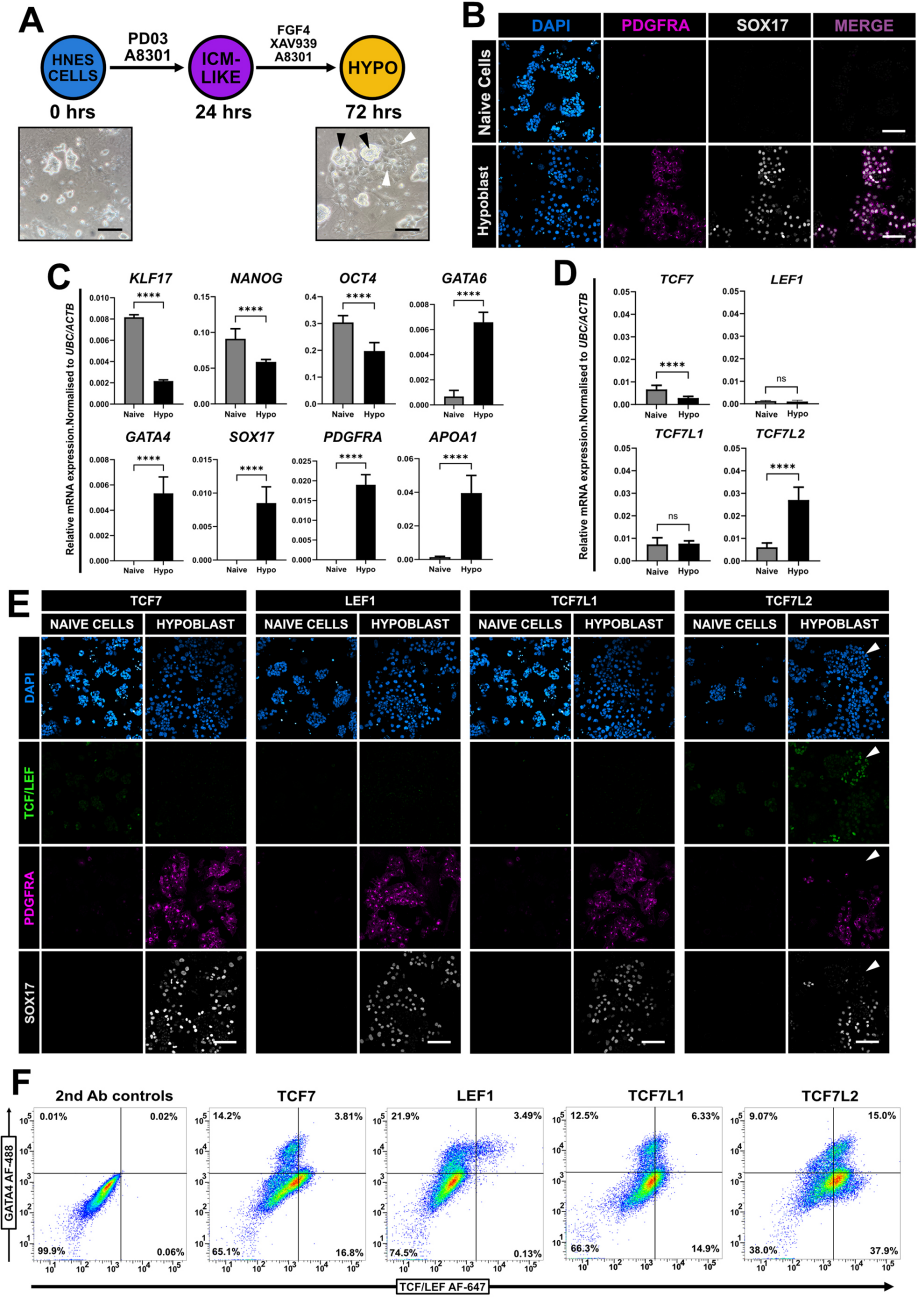
**TCF/LEF expression in hypoblast cells.** (A) Schematic of differentiation protocol to hypoblast-like cells from human naïve ES cells over 72 h. Brightfield images of HNES3 cells 24 h after plating (0 h) and hypoblast-like cells in differentiation conditions 72 h later are shown. Undifferentiated pluripotent colonies (black arrowheads) surrounded by nascent hypoblast-like cells (white arrowheads) are indicated. Scale bars: 100 μm. ICM, inner cell mass. (B) Immunofluorescence of undifferentiated HNES3 cells in PXGL (naïve cells) and differentiated hypoblast-like cells after 72 h, labelled for PDGFRA (magenta) and SOX17 (grey). Scale bars: 100 μm. Images in A and B are representative of five independent fields of view. (C) RT-qPCR analysis of pluripotency and hypoblast markers after 96 h either in PXGL (control, naïve cells) or differentiation (hypoblast-like cells, hypo) conditions. One-tailed Student's paired *t*-test (****P<0.0001). Mean±s.d., *N*=3. (D) RT-qPCR analysis of the TCF/LEF genes after 96 h either in PXGL (control) or differentiation conditions. One-tailed Student's paired *t*-test (****P<0.0001; ns, not significant). Mean±s.d., *N*=3. (E) Immunofluorescence of HNES3-derived hypoblast-like cells and control naïve cells labelled for each TCF/LEF factor (green), PDGFRA (magenta) and SOX17 (grey). Note increased TCF7L2 localisation (white arrowheads). Representative images from *N*=3 independent experiments. Scale bars: 100 μm. (F) Distribution plots of HNES3-derived hypoblast cells after 96 h in differentiation conditions labelled for each of the TCF/LEF factors and GATA4. Representative of at least *N*=3 independent experiments. 2nd Ab controls, secondary-antibody-only control.

To study TCF/LEF factors in human hypoblast development, we utilised a reproducible and simple two-dimensional differentiation protocol using a stepwise strategy primarily activating FGF signalling (Dattani et al., 2024) (Fig. 2A). After 72 h, patches of epithelised cells emerged, surrounding refractile colonies (Fig. 2A). Immunofluorescence confirmed co-expression of the hypoblast markers PDGFRA and SOX17 after 72 h but not in naïve ES cells (Fig. 2B). Downregulation of the naïve pluripotency markers *KLF17*, *NANOG* and *OCT4*, and an upregulation of *GATA6*, *GATA4* and *SOX17*, as well as the hypoblast-specific markers *PDGFRA* and *APOA1*, was validated using qPCR (Fig. 2C), confirming a hypoblast identity. Further qPCR analysis showed a strong upregulation of *TCF7L2*, whereas expression of *TCF7L1* and *LEF1* remained unchanged and *TCF7* was further reduced in hypoblast cells compared to naïve cells (Fig. 2D). Immunofluorescence of hypoblast cells indicated TCF7L2 expression was low in PDGFRA-positive SOX17-positive cells and more prominent in a population of cells that did not express PDGFRA and SOX17 (Fig. 2E). This observation suggests a persistent alternative population of cells expressing TCF7L2, which likely represents trophectoderm. Flow cytometry confirmed a small percentage of GATA4-expressing hypoblast cells after 72 h in differentiation conditions (Fig. 2F), which largely corroborated our immunofluorescence observations. Of note, a very small population of GATA4-positive LEF1-positive cells were present, which accounted for ∼2.5–3.5% of the total population (Fig. 2F).

We next compared these observations with naïve hPSCs treated with ACL cocktail (Activin A, CHIR99021 and LIF) for 5–6 days to generate naïve primitive endoderm (nEND) (Linneberg-Agerholm et al., 2019) or yolk sac-like cells (Mackinlay et al., 2021) that appear to represent a later stage of human development compared to hypoblast cells (Dattani et al., 2024). Therefore, we compared TCF/LEF factor and key marker gene expression between hypoblast cells and nEND. When naïve hPSCs were cultured in ACL for 5–6 days, naïve colonies collapsed, and widespread epithelialisation was observed (Fig. S2A). We then utilised qPCR to quantify mRNA transcripts in different culture conditions (Fig. S2B). *KLF17*, *NANOG* and *OCT4* expression were all strongly reduced in nEND cells and less strongly reduced in hypoblast cells, corroborating previous reports (Dattani et al., 2024; Linneberg-Agerholm et al., 2019). Although *GATA6* and *GATA4* expression remained relatively unchanged between each culture condition, we observed much higher levels of *PDGFRA* and *APOA1* in hypoblast cells. *SOX17* was undetectable in nEND cells; however, we could detect elevated levels of the mesoderm marker *APLNR* (Jackson et al., 2021; Lam et al., 2023; Sagrac et al., 2020) (Fig. S2B). Further analysis revealed that *TCF7L2* expression was clearly increased in hypoblast-inducing culture conditions, whereas *TCF7*, *LEF1* and *TCF7L1* were highly expressed in nEND, demonstrating a key difference between these cell populations (Fig. S2C). Immunofluorescence of hypoblast cells revealed no major differences in TCF/LEF signals (Fig. S2D), whereas in nEND cells, TCF7 was localised in both GATA4-positive and NANOG-positive nuclei, LEF1 was restricted to GATA4-positive cells, and TCF7L1 was co-expressed with small clusters of NANOG-positive pluripotent cells (Fig. S2E).

Collectively, these observations reveal differences in the expression of the four TCF/LEF factors that might reflect the distinct developmental stages and status of WNT signalling (activation versus inhibition) when inducing different populations of hypoblast and nEND cells.

### Naïve hPSC-derived trophectoderm expresses high levels of TCF7L2

**Fig. 3.**
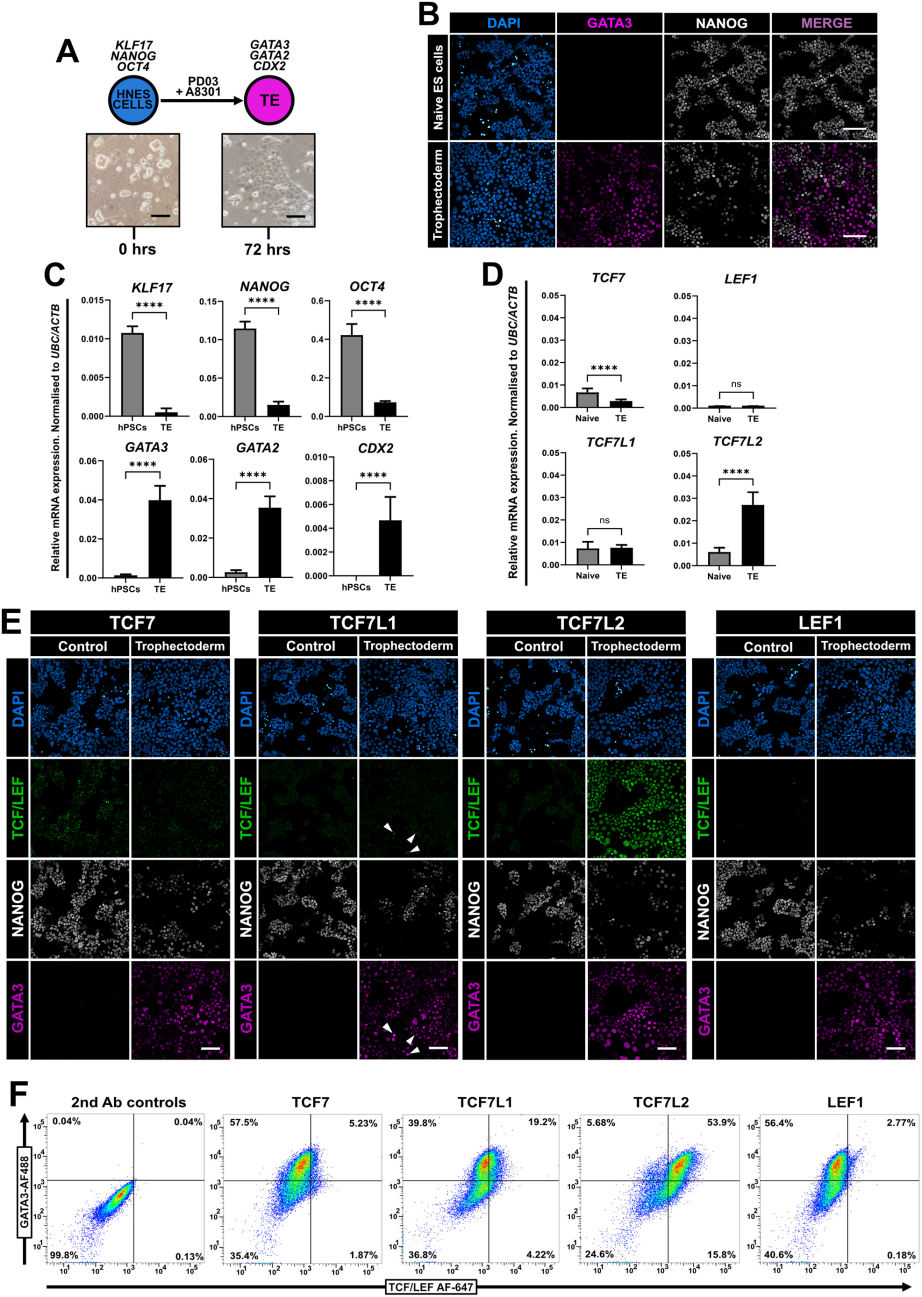
**Trophectoderm cells express high levels of TCF7L2.** (A) Schematic of differentiation protocol to trophoblast-like cells from HNES cells using PD0325901 and A8301 for 72 h. Brightfield images of HNES1 cells at 24 h after plating and 72 h later in differentiation conditions are shown. Scale bars: 100 μm. Images are representative of five independent fields of view. (B) Immunofluorescence of undifferentiated cR-H9 cells in PXGL (naïve control) and differentiated trophectoderm cells after 72 h in PDA83. Cells are labelled for NANOG (grey) and GATA3 (magenta). Scale bars: 100 μm. Representative of three fields of view and *N*=3 independent experiments. (C) RT-qPCR analysis of pluripotent and trophectoderm marker genes in control cells (hPSCs) and after 72 h in differentiation medium (TE). One-tailed Student's paired *t*-test (****P<0.0001). Mean±s.d., *N*=3. (D) RT-qPCR analysis of TCF/LEF genes in control cells (naïve) and after 72 h in differentiation medium (TE). One-tailed Student's paired *t*-test (****P<0.0001; ns, not significant). Mean±s.d., *N*=3. (E) Immunofluorescence of HNES3 cells cultured in PXGL (control) and trophectoderm cells after 72 h in differentiation medium. Cells are labelled for NANOG (grey), TCF/LEF factors as indicated (green) and GATA3 (magenta). White arrowheads represent TCF7L1 nuclei that also express GATA3. Note overlap of cells co-expressing TCF7L2 and either GATA3 or NANOG. Scale bars: 100 μm. *N*=3. (F) Flow cytometric analysis of naïve ES and trophectoderm cells at 96 h labelled for each TCF/LEF factor and GATA3. *N*=3. 2nd Ab controls, secondary-antibody-only control.

Naïve hPSCs can differentiate into trophectoderm cells in differentiation medium PDA83, which contains PD0325901 (an inhibitor of MEK–ERK signalling) and A8301 (a potent inhibitor of TGFβ–NODAL signalling) (Guo et al., 2021; Io et al., 2021). Naïve hPSCs cells were plated onto Geltrex-coated plates and switched to PDA83 for 72 h (Fig. 3A). Characteristic patches of epithelised cells emerged, and immunofluorescence confirmed GATA3 expression (trophectoderm) and downregulation of NANOG (pluripotency) (Fig. 3B). *KLF17*, *NANOG* and *OCT4* mRNA levels decreased over 72 h in PDA83, with a concomitant strong increase in expression of the trophectoderm markers *GATA3, GATA2* and *CDX2* (Fig. 3C). qPCR analysis revealed reduced levels of *TCF7*, no change in *TCF7L1* or *LEF1*, and a strong increase in *TCF7L2* expression in trophectoderm (Fig. 3D)*.* Further immunofluorescence of trophectoderm cultures at 72 h confirmed our initial qPCR observations, including low and heterogeneous TCF7L1-positive GATA3-positive cells and TCF7L2 nuclear signal in both NANOG-positive and GATA3-positive cells (Fig. 3E). Flow cytometry of trophectoderm revealed a small population of TCF7L1-positive GATA3-positive cells (∼20%), and two populations of cells expressing TCF7L2 with GATA3 (∼53%) and without GATA3 (∼16%) were present in these cultures (Fig. 3F). Taken together, these data indicate that TCF7L2 is a previously uncharacterised marker of human trophectoderm and is upregulated in response to simultaneous inhibition of MEK–ERK and NODAL signalling.

### TCF/LEF factor expression in human blastocysts and blastoid models

**Fig. 4.**
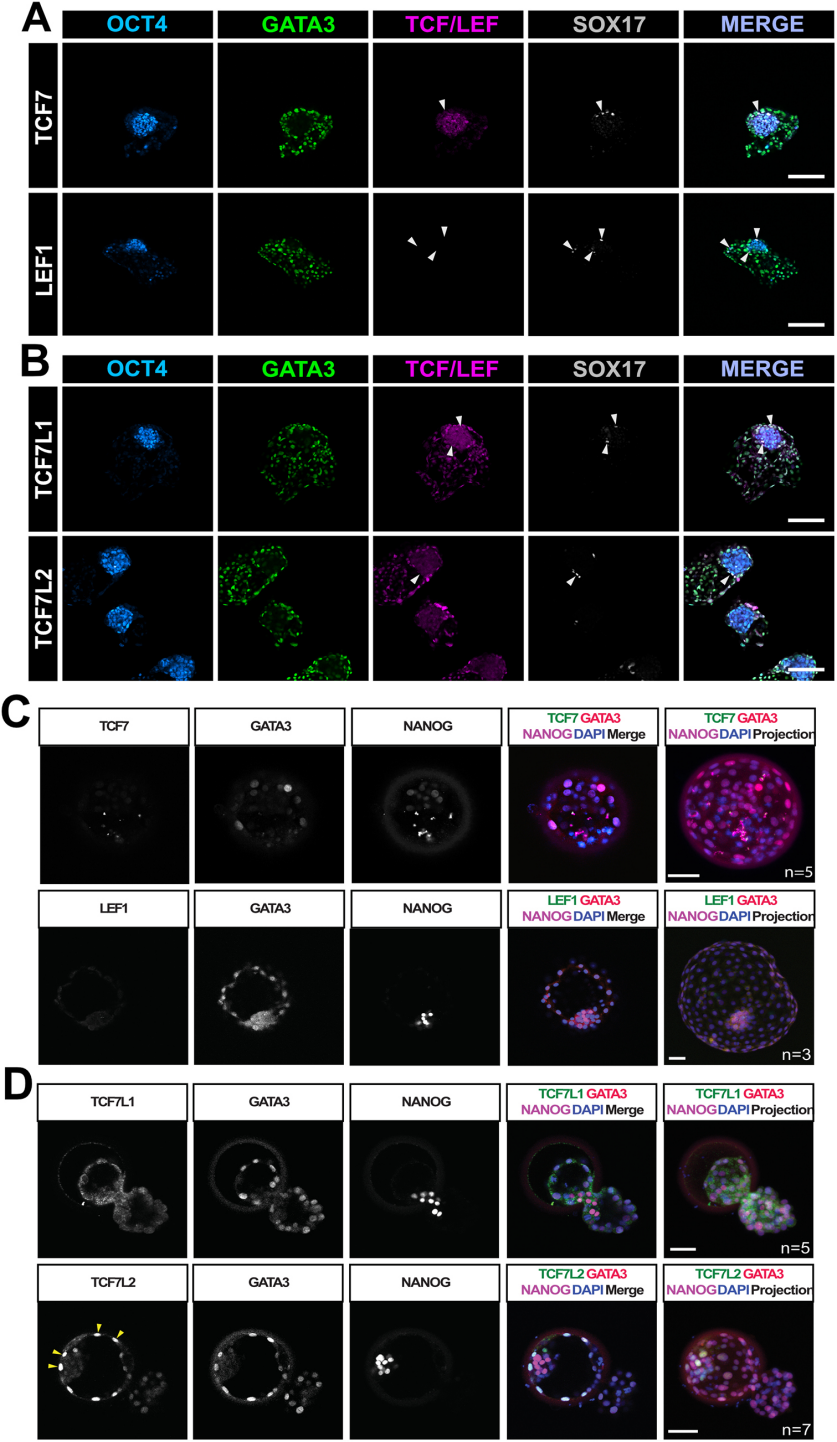
**TCF/LEF factor expression in human blastocysts and blastoid models.** (A) Immunofluorescence of HNES1-derived blastoids for OCT4 (cyan), GATA3 (green), the indicated TCF7/LEF1 protein (magenta) and SOX17 (grey) after 96 h of culture. Scale bars: 100 μm. Arrowheads indicate SOX17-positive hypoblast-like cells. *N*=7 blastoids per panel. (B) Immunofluorescence of HNES1-derived blastoids for OCT4 (cyan), GATA3 (green), TCF7L1 or TCF7L2 (magenta), and SOX17 (grey) after 96 h of culture. Scale bars: 100 μm. Arrowheads indicate SOX17-positive hypoblast-like cells. *N*=8 blastoids per panel. (C) Representative images of immunofluorescence analysis of TCF7 and LEF1 (green), GATA3 (red), NANOG (magenta) and DAPI nuclear staining (blue) in day 6 post-fertilisation human blastocysts, along with sum slice projections. The number of embryos analysed is indicated. Scale bars: 50 μm. (D) Representative images of immunofluorescence analysis of TCF7L1 and TCF7L2 (green), GATA3 (red), NANOG (magenta) and DAPI nuclear staining (blue) in day 6 post-fertilisation human blastocysts, along with sum slice projections. The number of embryos analysed is indicated. Scale bars: 50 μm. Yellow arrowheads indicate TCF7L2-positive GATA3-positive trophectoderm nuclei.

To assess TCF/LEF expression in a multi-tissue context, we generated blastoids, three-dimensional stem cell-based models of early human development (Fig. S3A) (Kagawa et al., 2022; Yanagida et al., 2021). Immunofluorescence revealed TCF7 signal in the nuclei of OCT4-positive epiblast-like cells and LEF1 was undetectable (Fig. 4A). In contrast to our early observations in naïve-derived trophectoderm cells, we could readily detect TCF7L1 in the nuclei of GATA3-positive trophectoderm-like cells, with a haze of signal in the OCT4-positive epiblast-like compartment (Fig. 4B). GATA3-positive trophectoderm-like cells also stained strongly for TCF7L2 (Fig. 4B), in accordance with our two-dimensional trophectoderm cells (see Fig. 3E). To corroborate our observations, we performed immunofluorescence on preimplantation human blastocysts (Fig. 4C,D). TCF7 and LEF1 were undetectable in any of the three founding lineages (epiblast, hypoblast and trophectoderm; Fig. 4C), despite blastoids exhibiting strong nuclear TCF7 in the epiblast-like cells (Fig. 4A). Nuclear-localised TCF7L1 signal was absent in GATA3-positive trophectoderm, whereas strong TCF7L2 was reproducibly detected in these cells in human blastocysts (Fig. 4D) but absent in the epiblast, corroborating our blastoid observations.

Next, we accessed publicly available integrated transcriptomics of human embryo development (Zhao et al., 2025) (Fig. S3B), providing a comprehensive database to interrogate gene expression signatures based on previous lineage annotations (Fig. S3C). Although *LEF1* is not expressed in any preimplantation lineage, *TCF7* is widely expressed in the epiblast, hypoblast and trophectoderm at different development stages (Fig. S3D), which does not align with our blastoid or human blastocyst immunofluorescence data. *TCF7L1* expression shows enrichment primarily in the trophectoderm lineages (Fig. S3E), which we observed in blastoid models but not human blastocysts. In contrast, *TCF7L2* expression is present in the hypoblast and trophectoderm lineages, similar to both our blastoid and human embryo immunofluorescence data (Fig. 4B,D). We also interrogated the expression of two well-characterised WNT signalling readout genes, *AXIN2* (Jho et al., 2002) and *SP5* (Fujimura et al., 2007; Huggins et al., 2017; Kennedy et al., 2016) (Fig. S3F). *AXIN2* expression appears enriched in later trophectoderm lineages (cytotrophoblast and syncytiotrophoblast), supporting previously reported roles for WNT signalling in these lineages in human (Dietrich et al., 2022; Shukla et al., 2024) and non-human primates (Matsumoto and Tanaka, 2024; Siriwardena et al., 2024), and the epiblast. *SP5* expression is restricted to the early hypoblast, mesoderm and definitive endoderm lineages, with little overlap with *AXIN2* (Fig. S3F).

Taken together, these data demonstrate that two- and three-dimensional stem-cell-derived models can recapitulate aspects of human embryo development; however, relatively minor differences can be detected. Moreover, we show, for the first time, TCF7L2 expression in the human trophectoderm lineages, which encourages further investigation into its role in controlling trophectoderm development, cell differentiation and implantation.

### Active WNT/β-catenin signalling induces *TCF7* and *LEF1* in primed pluripotent cells

**Fig. 5.**
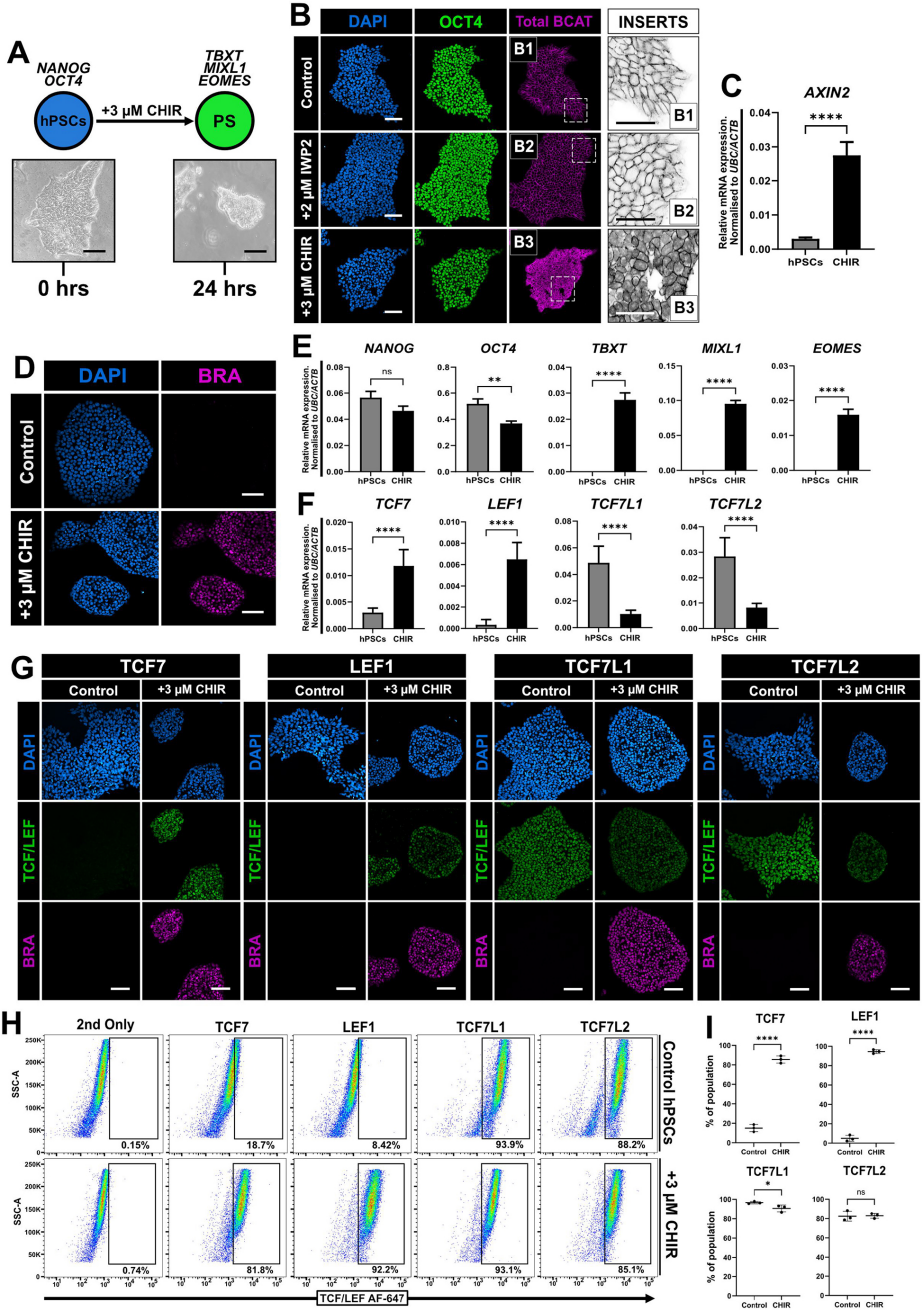
**TCF7 and LEF1 are upregulated in response to WNT activation in human primed pluripotent ES cells.** (A) Schematic of primed human pluripotent embryonic cells (ES cells and induced pluripotent stem cells) cultured in StemFlex then switched to N2B27 with 3 µM CHIR (WNT activator) for 24 h (PS, primed state). Brightfield images representative of *N*=5 cell lines are shown. Scale bars: 100 μm. (B) Immunofluorescence for β-catenin (BCAT) and OCT4 (green) in capacitated HNES1 cells cultured in StemFlex medium for 72 h following passaging with and without 2 µM IWP2 (WNT inhibitor) or switched to N2B27 with 3 µM CHIR for 24 h. Scale bars: 100 μm. Insert images of regions marked by dashed boxes for B1, B2 and B3 are representative of β-catenin labelling in each condition. Insert scale bars: 200 µm. *N*=3. (C) RT-qPCR analysis of *AXIN2* expression in control primed ES cells (hPSCs) versus cells treated with 3 µM CHIR for 24 h. Relative mRNA levels were normalised to *UBC* and *ACTB*. One-tailed Student's paired *t*-test (****P<0.0001). Mean±s.d. *N*=3. (D) Immunofluorescence of capacitated HNES1 cells in control and differentiation conditions (N2B27+3 µM CHIR for 24 h), labelled for BRACHYURY. Scale bars: 100 μm. *N*=3. (E) RT-qPCR analysis of control cells (hPSCs) and treated primed cells (CHIR) for *NANOG*, *OCT4*, *TBXT*, *MIXL1* and *EOMES*. Relative mRNA levels were normalised to *UBC* and *ACTB*. One-tailed Student's paired *t*-test (ns, not significant; **P<0.01; ****P<0.0001). Mean±s.d. *N*=3. (F) RT-qPCR analysis of control cells (hPSCs) and treated primed cells (CHIR) for *TCF7*, *LEF1*, *TCF7L1* and *TCF7L2*. Relative mRNA levels were normalised to *UBC* and *ACTB*. One-tailed Student's paired *t*-test (****P<0.0001). Mean±s.d., *N*=3. (G) Immunofluorescence of control H9 cells and H9 cells treated with N2B27+3 µM CHIR for 24 h, labelled for each TCF/LEF (green) and BRACHYURY (magenta). Representative images for *N*=5 independent experiments. Scale bars: 100 μm. (H) Flow cytometric analysis of control and treated (N2B27+3 µM CHIR for 24 h) primed cells labelled for each TCF/LEF factor. Percentages of cells within the marked TCF/LEF-positive gates are shown. 2nd only, secondary-antibody-only control. *N*=3. (I) Quantification of TCF/LEF-expressing cells in hPSC control and differentiation conditions (CHIR) after 24 h. One-tailed Student's paired *t*-test (*P<0.05; ****P<0.0001; ns, not significant). Mean±s.d., *N*=3.

WNT/β-catenin signalling is essential for the initiation of gastrulation and, subsequently, the differentiation of mesendoderm cells into mesodermal and endodermal subtypes (Barrow et al., 2007; Biechele et al., 2013; Liu et al., 1999). Primed hPSCs in undifferentiated conditions express TCF7L1 and TCF7L2, but neither TCF7 nor LEF1 (Fig. 1F), which is in agreement with previous observations (Sierra et al., 2018) and in stark contrast to mouse primed cells (Fig. 1C). We then asked whether activation of WNT/β-catenin signalling initiating primitive streak/mesendoderm differentiation would change TCF/LEF expression. hPSCs were treated with either CHIR or WNT3A for 24 h to initiate primitive streak differentiation. Indeed, upon CHIR or WNT3A treatment, colonies of hPSCs formed domes with refractile edges (Fig. 5A) and β-catenin was localised to the nuclei of cells (Fig. 5B; Fig. S4A). Concomitant with a change in cell morphology, β-catenin was present in the nuclei of CHIR-treated (WNT activator) but not control or IWP2-treated (WNT inhibitor) hPSCs, similarly to previous reports in hPSCs (Dziedzicka et al., 2021; Massey et al., 2019; Sierra et al., 2018), colorectal cancer cells (Ambrosi et al., 2022) and HEK293T cells (Kafri et al., 2016). To monitor activation of the WNT/β-catenin pathway, we measured expression of the WNT target gene *AXIN2* (Jho et al., 2002), which showed a strong upregulation when hPSCs were treated with CHIR and to a lesser extent with WNT3A (Fig. 5C; Fig. S4B). Furthermore, immunofluorescence established the presence of widespread BRACHYURY (BRA, encoded by *TBXT*) expression, a target gene of WNT signalling (Arnold et al., 2000; Yamaguchi et al., 1999) (Fig. 5D; Fig. S4B,C), with increased levels of *TBXT, MIXL1* and *EOMES* mRNA, strongly when cells were treated with CHIR (Fig. 5E) and modestly with WNT3A recombinant protein (Fig. S4B). Upon differentiation, the expression profiles of all four TCF/LEF genes changed, with expression of both *TCF7L1* and *TCF7L2* being strongly reduced alongside an increase in *TCF7* and *LEF1* mRNA levels (Fig. 5F). There was a more pronounced effect on the expression of the TCF/LEF factors with CHIR treatment than with WNT3A (Fig. S4D). Immunofluorescence of TCF/LEF expression in control and treated hPSCs largely corroborated our initial observations with qPCR (Fig. 5G; Fig. S4E). Interestingly, we observed LEF1-positive nuclei around the edge of the colony following WNT3A but not following CHIR treatment (Fig. S4D), similar to a previous report using micropatterned gastruloids (Martyn et al., 2019). Finally, flow cytometric analysis of CHIR-treated hPSCs demonstrated a strong increase in both TCF7-positive (∼82%) and LEF1-positive (∼92%) cells, whilst TCF7L1- and TCF7L2-expressing cell numbers were slightly decreased (Fig. 5H,I). Of note, despite the total number of gated cells increasing only slightly, there was an observable shift in their overall signal intensity (Fig. 5H).

Collectively, these results on TCF/LEF expression in hPSCs clearly differ from the mouse paradigm. In human primed pluripotent cells, we show that alongside LEF1 (Funa et al., 2015; Martyn et al., 2019), TCF7 is also upregulated in response to active WNT signalling. Moreover, both *TCF7L1* and *TCF7L2* are subsequently downregulated as differentiation proceeds, perhaps to control the spatiotemporal expression of mesendoderm genes (Hoffman et al., 2013; Sierra et al., 2018).

### TCF7L1 and TCF7L2 expression persists in amnion cells

**Fig. 6.**
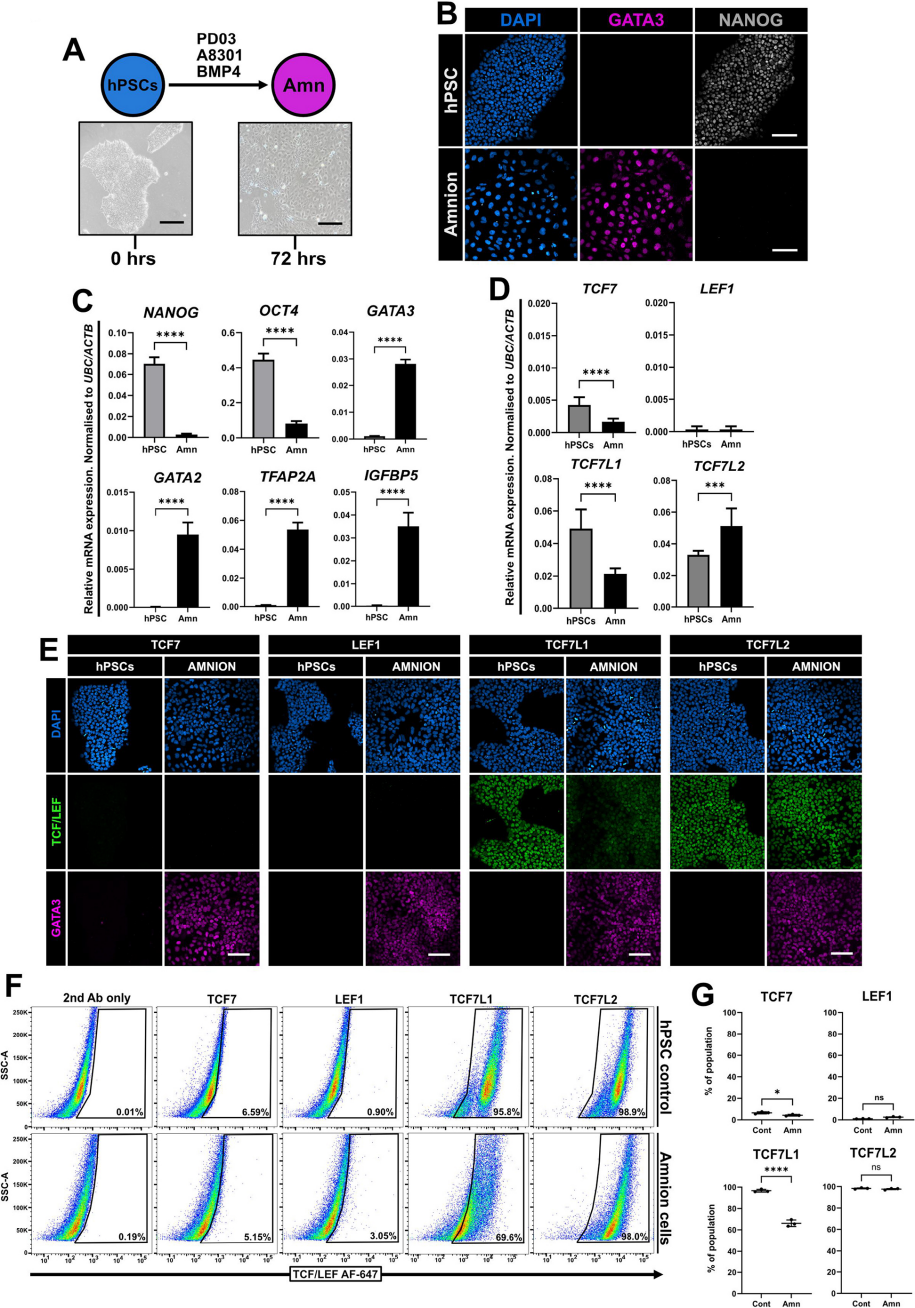
**TCF7L1 and TCF7L2 expression in amnion differentiation.** (A) Experimental protocol for amnion (Amn) differentiation. Phase-contrast images of primed HNES3 (pHNES3) control hPSCs and amnion cells at 72 h of differentiation. Scale bars: 100 μm. Images are representative of five independent fields of view. (B) Immunofluorescence of control pHNES3 and amnion cells after 72 h in PDA83+BMP4 for 72 h. Cells are labelled for GATA3 (magenta) and NANOG (grey). Scale bars: 100 μm. *N*=3. (C) RT-qPCR of pluripotency and amnion markers from control (hPSC) and differentiated primed (Amn) cell lines. One-tailed Student's paired *t*-test (****P<0.0001). Mean±s.d., *N*=3. (D) RT-qPCR of TCF/LEF factors in undifferentiated and amnion cells after 72 h in differentiation conditions. One-tailed Student's paired *t*-test (***P<0.001; ****P<0.0001). Mean±s.d., *N*=3. (E) Immunofluorescence of control hPSCs and differentiated amnion cells labelled for the indicated TCF/LEF factors (green) and GATA3 (magenta). Scale bars: 100 μm. *N*=3. (F) Flow cytometry of control hPSCs and amnion cells labelled for each of the TCF/LEF factors, as indicated, after 72 h. Percentages of cells within the marked TCF/LEF-positive gates are shown. 2nd Ab only, secondary-antibody-only control. *N*=3. (G) Quantification of TCF/LEF-positive cells in control conditions (Cont) and after 72 h of differentiation (Amn). One-tailed Student's paired *t*-test (*P<0.05; ****P<0.0001; ns, not significant). Mean±s.d., *N*=3.

Activation of BMP signalling in primed hPSCs has been reported to induce their differentiation into either trophoblast (Amita et al., 2013; Roberts et al., 2014; Xu et al., 2002; Yabe et al., 2016) or amnion cells (Guo et al., 2021; Io et al., 2021; Rostovskaya et al., 2022). The embryonic relationship between these tissues remains an area of controversy, but recent data suggest that primed hPSCs generate amnion, whereas naïve hPSCs generate trophectoderm (Guo et al., 2021; Io et al., 2021; Rostovskaya et al., 2022). To induce amnion differentiation, cells were treated for 72 h with PD0325901, A8301 and BMP4, where compact colonies of primed hPSCs transitioned to flattened epithelial sheets (Fig. 6A), which were GATA3-positive and NANOG-negative (Fig. 6B). *NANOG* and *OCT4* expression levels decreased, and expression of *GATA3*, *GATA2*, *TFAP2A* and *IGFBP5* were strongly increased (Fig. 6C), denoting entry into the amnion lineage (Io et al., 2021). Expression levels of both *TCF7* and *TCF7L1* were decreased, *TCF7L2* expression subsequently increased and *LEF1* expression was unchanged (Fig. 6D). Immunofluorescence of control and differentiated cells revealed no signal for TCF7 or LEF1. TCF7L1 signal appeared to be diminished in amnion cells compared to control hPSCs, although TCF7L2 signal did not change (Fig. 6E). We employed flow cytometry to measure the changes in protein expression between hPSC controls and amnion at 72 h of differentiation. The incidence of TCF7L1-positive cells decreased and the number of TCF7L2-positive cells showed no difference (Fig. 6F,G). Extensive alternative splicing of *TCF7L2* has been shown to produce a wide range of functionally diverse proteins with varying functional properties (reviewed by Hoppler and Waterman, 2014; Weise et al., 2010) and may account for the discrepancy between detected mRNA and protein levels.

Collectively, these data suggest that reduced TCF7L1 levels and persistence of TCF7L2 could be an important prerequisite during primate amnion development in defining an epiblast–amnion boundary (Bergmann et al., 2022; Rostovskaya et al., 2022) and shield against active WNT/β-catenin signalling induced by BMP4 signalling that would otherwise drive gastrulation (Martyn et al., 2019; Yoney et al., 2022).

### Temporal expression of the TCF/LEF factors in definitive endoderm

**Fig. 7.**
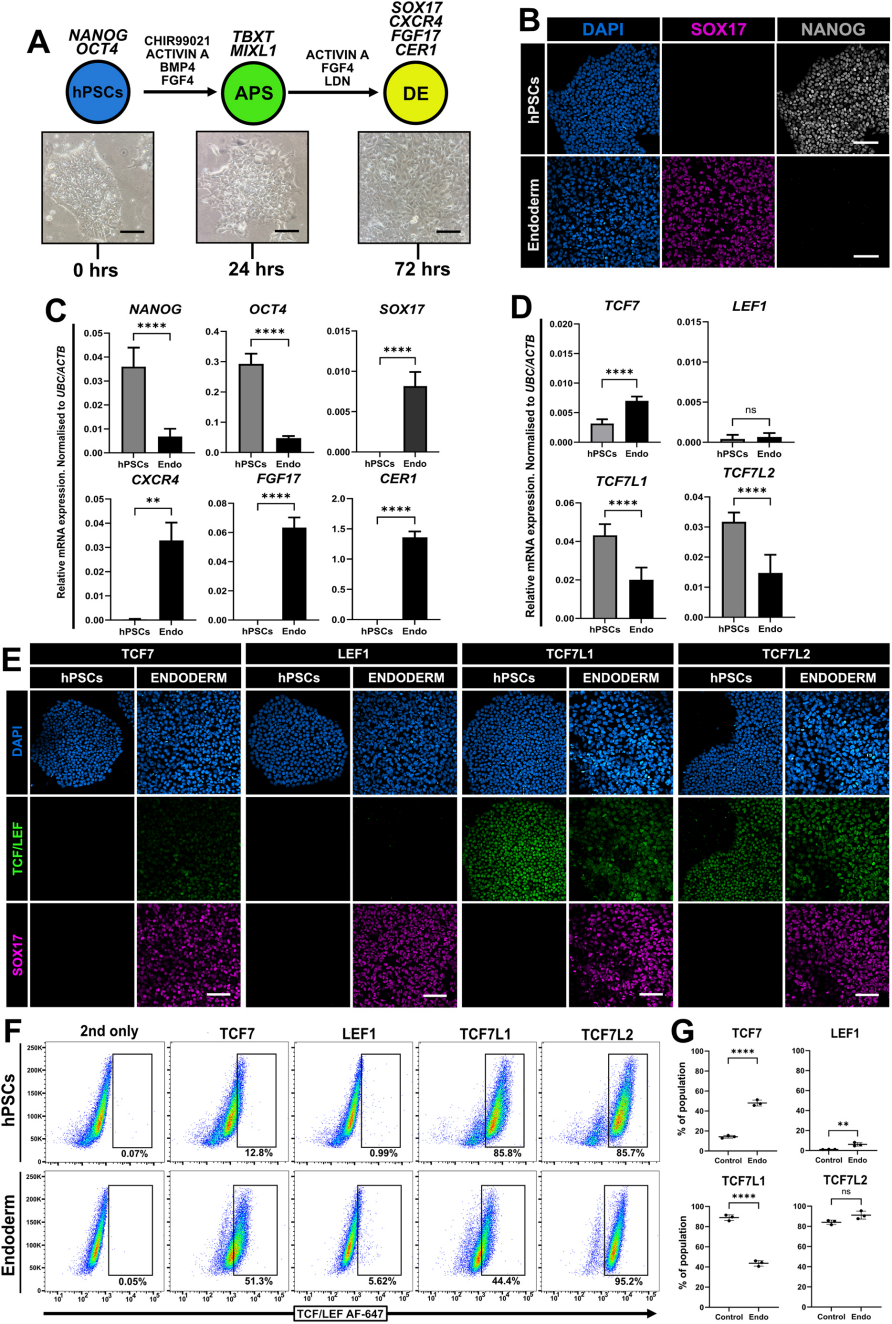
**TCF/LEF factor expression in definitive endoderm from hPSCs.** (A) Experimental schematic to differentiate hPSCs towards definitive endoderm (DE). APS, anterior primitive streak. Brightfield images show primed HNES3 (pHNES3) control hPSCs and DE cells. Scale bars: 100 μm. Images are representative of five independent fields of view. (B) Immunofluorescence of control H9 cells (hPSCs) and definitive endoderm after 72 h of differentiation. Cells are labelled for SOX17 (magenta) and NANOG (grey). Scale bars: 100 μm. *N*=3. (C) RT-qPCR analysis of control hPSCs and definitive endoderm (Endo) at 72 h. Relative mRNA levels were normalised to *UBC* and *ACTB*. One-tailed Student's paired *t*-test (**P<0.01; ****P<0.0001). Mean±s.d., *N*=3. (D) RT-qPCR analysis of control hPSCs and definitive endoderm (Endo) at 72 h for *TCF7*, *TCF7L1*, *TCF7L2* and *LEF1*. Relative mRNA levels were normalised to *UBC* and *ACTB*. One-tailed Student's paired *t*-test (ns, not significant; ****P<0.0001). Mean±s.d., *N*=3. (E) Immunofluorescence of pHNES3 hPSCs and definitive endoderm after 72 h of differentiation. Cells are labelled for SOX17 (magenta) and each TCF/LEF factor (green), as indicated. Scale bars: 100 μm. *N*=3. (F) Flow distribution plots of control hPSCs and definitive endoderm after 72 h of differentiation. Percentages of cells within the marked TCF/LEF-positive gates are shown. 2nd only, secondary-antibody-only control. *N*=3. (G) Quantification of TCF/LEF-positive cells in hPSCs (Control) and definitive endoderm (Endo) samples after 72 h. Mean±s.d., *N*=3. One-tailed Student's paired *t*-test (ns, not significant; **P<0.01; ****P<0.0001).

Shortly following the formation of the primitive streak and mesendoderm cells in the mammalian embryo (Rivera-Perez and Magnuson, 2005), fine-tuning of BMP, NODAL, WNT and FGF signalling facilitates further differentiation into mesodermal subtypes and definitive endoderm (Bardot and Hadjantonakis, 2020; Bergmann et al., 2022; Tam and Behringer, 1997; Tyser et al., 2021). We leveraged a robust protocol to efficiently differentiate primed hPSCs in a stepwise fashion towards definitive endoderm cells (Loh et al., 2014) and subsequently profiled the expression of the TCF/LEF factors. hPSCs were treated with signalling modulators over 72 h, which gave rise to a monolayer of epithelial cells (Fig. 7A). Whilst SOX17 (endoderm) was widely visible, NANOG (pluripotency) was lost (Fig. 7B). Pluripotency makers *NANOG* and *OCT4* were downregulated, and expression of canonical endoderm markers *SOX17*, *CXCR4*, *FGF17* and *CER1* was strongly increased at 72 h (Fig. 7C). Further analysis revealed no significant changes in *LEF1* expression; however, *TCF7* expression was increased and both *TCF7L1* and *TCF7L2* expression levels were lower than in control hPSCs (Fig. 7D). Immunofluorescence confirmed widespread SOX17-positive endoderm cells with visible TCF7, TCF7L1 and TCF7L2 signal, but no expression of LEF1 (Fig. 7E). Flow cytometric analysis revealed a population of TCF7-positive cells (∼51%), TCF7L1-positive cells decreased and whilst the total numbers of TCF7L2-positive cells exhibited no significant difference, the overall intensity of this signal shifted (Fig. 7F,G).

These findings suggest that both TCF7 and LEF1 exhibit temporal activity whereby their expression decreases over time during definitive endoderm commitment following initial upregulation in primed hPSCs following WNT stimulation. Moreover, despite the strong decreases in the mRNA levels of *TCF7L1* and *TCF7L2* in SOX17-positive endoderm, their expression suggests a role in maintaining lineage identity whilst preventing precocious differentiation into other cell types.

## DISCUSSION

Our current understanding of WNT signalling during early mammalian development and stem cell biology has been predominantly informed by rodent models. However, applying this rodent paradigm to early human development has become increasingly difficult. Here, we leveraged mouse and hPSC models to test conservation of expression and localisation of TCF7, LEF1, TCF7L1 and TCF7L2, the canonical terminal effectors of the WNT/β-catenin pathway (Fig. S5).

### Differences in states of pluripotency

We show that mouse and human pluripotent stem cells comprehensively differ in their expression of TCF/LEF factors (Fig. 1). We confirm in mESCs the expression of all four TCF/LEF genes but find only low levels of TCF7L2 in human naïve pluripotency. It is interesting to find heterogeneous Lef1 expression in the mouse (Fig. 1C); since Lef1 expression is known to increase upon release from naïve pluripotency (Ye et al., 2017), this Lef1-positive subpopulation may represent those cells ready to exit the naïve pluripotency state. Consistent with that, in mouse primed pluripotency (i.e. mEpiSCs; Fig. 1C–E), we reproducibly detected Lef1 expression across multiple lines, mirroring E7.5 stage embryos (Galceran et al., 2001; Oosterwegel et al., 1993). In primed hPSCs, in contrast to the mouse, we detect essentially no LEF1 expression (Fig. 1F–H, see also Fig. 5G). These species-specific differences in TCF/LEF expression are likely related to the revealed different requirements for WNT/β-catenin signalling between mouse and human for stable propagation of the naïve pluripotent state (Bayerl et al., 2021; Bredenkamp et al., 2019; Guo et al., 2017; Lyashenko et al., 2011; Takashima et al., 2014; Wray et al., 2011; Ying et al., 2008). Culture conditions mirroring mouse 2i+LIF are unsuitable for stable propagation of naïve hPSCs (Gafni et al., 2013; Hanna et al., 2010; Takashima et al., 2014). Reducing WNT activation by titrating the concentration of CHIR improves propagation of naïve hPSCs (Takashima et al., 2014; Theunissen et al., 2014). More recently, it has been shown that exchanging CHIR (WNT activator) with a potent tankyrase inhibitor, XAV939 (initially described as a Wnt inhibitor, Huang et al., 2009), improves self-renewal (Bayerl et al., 2021; Bredenkamp et al., 2019; Guo et al., 2017; Khan et al., 2021; Zimmerlin et al., 2016) but predominantly by buffering Hippo signalling (Dattani et al., 2022). Consistent with only low expression of TCF7 and TCF7L2, naïve hPSCs lacking functional β-catenin protein maintain expression of key naïve pluripotent markers, implying that the WNT/β-catenin pathway may be largely dispensable for propagating human naïve pluripotency (Bayerl et al., 2021; Dattani et al., 2022).

Despite its very low expression in naïve hPSCs, *TCF7L1* is gradually upregulated towards the later stages of capacitation (Rostovskaya et al., 2019) and is homogenously expressed in primed hPSCs, raising several fundamental questions regarding its role in the regulation of pluripotency in mammals (Fig. 1F–H; see also Sierra et al., 2018). These lower levels of TCF7L1 might contribute to the unrestricted lineage potential of naïve hPSCs (Dattani et al., 2024; Guo et al., 2021; Linneberg-Agerholm et al., 2019), even though mouse Tcf7l1 does not restrict differentiation of all embryonic cell lineages (e.g. primitive endoderm, Athanasouli et al., 2023); this deserves further investigation. Moreover, TCF7L1 may play a key and currently undefined role in primate development to influence the developmental pace of the epiblast lineage, which has been well-studied in the mouse previously (Hoffman et al., 2013; Pereira et al., 2006; Rostovskaya et al., 2019). Given its principal role as a strong repressor of gene expression (Kim et al., 2000; Liu et al., 2005; Yi et al., 2011), TCF7L1 essentially functions to tightly control differentiation and other signalling pathways, one of which is the NODAL pathway (Sierra et al., 2018). Both naïve and primed hPSCs require active NODAL signalling, and its inhibition downregulates key pluripotency markers such as *NANOG* (James et al., 2005; Osnato et al., 2021; Vallier et al., 2005). Moreover, when combined with blocking MEK–ERK signalling, naïve hPSCs readily undergo trophectoderm differentiation (Guo et al., 2021; Io et al., 2021). Conversely, in primed cells, TCF7L1 acts as a rheostat for a plethora of key gastrulation genes, and its knockdown drastically increases *NODAL* expression (Sierra et al., 2018). Therefore, lower levels of TCF7L1 might permit high *NODAL* expression necessary to support the long-term self-renewal of naïve hPSCs.

Since we have uncovered fundamental differences in human pluripotent cells between naïve and primed pluripotency, a future detailed analysis during capacitation is required. In support of this, studies of the transition from naïve to primed pluripotency have revealed a fundamental requirement for WNT/β-catenin inhibition to circumvent precocious differentiation (Rostovskaya et al., 2019). Thus, it is of interest to understand how the epiblast transitions between pluripotent states is shielded from WNT/β-catenin signalling, which could rely on SFRP family members secreted from the nascent epiblast (*SFRP1 and SFRP2*) and hypoblast (*FRZB*, also known as *SFRP3*) (Blakeley et al., 2015; Stirparo et al., 2018). Furthermore, while we compared mouse and human in what is currently widely considered comparable stages of pluripotency (naïve and primed), our findings here provide further evidence that they might not be equivalent in mouse and human. Mouse and human naïve pluripotent stem cells developmentally recapitulate the preimplantation epiblast of the embryo (Boroviak et al., 2014; Guo et al., 2016; Takashima et al., 2014), whereas mouse EpiSCs represent a mid-gastrula stage (Brons et al., 2007; Kinoshita et al., 2021; Kojima et al., 2014; Tesar et al., 2007; Tsakiridis et al., 2014), as compared to primed hPSCs, which resemble the pre-gastrula epiblast (Tyser et al., 2021; Xiang et al., 2020).

### Extra-embryonic and early embryonic cell lineages

A comparison of hypoblast cells (Dattani et al., 2024) with nEND cells (Linneberg-Agerholm et al., 2019) reveals clear differences in that hypoblast cells express only low levels of TCF7L2, whereas nEND differentiation upregulates *TCF7* and *LEF1* expression. Interestingly, following the nEND protocol, we readily detect upregulation of *APLNR*, which is usually considered a key mesoderm receptor involved in cardiac development (Jackson et al., 2021; Lam et al., 2023; Sagrac et al., 2020). In our hands, we observed a very small fraction of GATA4-positive LEF1-positive cells in hypoblast cultures. Although the embryonic origin of the extra-embryonic mesoderm (ExMe) remains unclear, a hypoblast origin in the peri-implantation primate embryo has recently been proposed (Ross and Boroviak, 2020). Consistent with this notion, data analysis from Bergmann et al. (2022) shows high *LEF1* expression in cells demarcated to be ExMe from single-cell RNA-sequencing data of peri/postimplantation marmoset embryos. Taken together, our data suggest that the two different culture conditions induce separate populations of differentiated cells that are likely to recapitulate hypoblast cells of the pre- and post-implantation human embryo (Dattani et al., 2024; Linneberg-Agerholm et al., 2019; Mackinlay et al., 2021). These differences in culture conditions could account for the contrasting TCF/LEF expression patterns in our current observations and might be related to the status of endogenous WNT signalling (i.e. WNT activation in nEND and inhibition in hypoblast cells).

Strikingly, we report here elevated TCF7L2 in trophectoderm cells derived from naïve hPSCs. We extended these observations and show TCF7L2 expression in GATA3-positive cells of both naïve hPSC-derived blastoids and preimplantation human blastocysts. TCF7L2 has known functional links in the regulation of metabolism (Verma et al., 2022), controlling context-specific cell differentiation (Lipiec et al., 2020) and proliferation (Wenzel et al., 2020). Moreover, the *TCF7L2* gene encodes alternatively spliced transcripts (Hoppler and Waterman, 2014; Torres-Aguila et al., 2022) that encode this diverse functionality of TCF7L2 protein isoforms (Young et al., 2019). By controlling trophoblast cell differentiation, TCF7L2 might ultimately have a role in human embryo implantation and subsequent trophoblast development. Intriguingly, the WNT inhibitor DKK1 is upregulated in the endometrium of women who have been hyper-stimulated with oestrogen/progesterone prior to egg collection, thereby reducing pregnancy rates (Liu et al., 2010). These increased levels of DKK1 might disrupt the embryonic–endometrial interface that is required normally to facilitate proper implantation. Our results suggest that TCF7L2 is upregulated in response to trophectoderm lineage induction and that heterogenous TCF7L1 expression might reflect a mixture of different trophoblast subtypes (Pollheimer et al., 2006). Whether *TCF7L2* expression precedes *GATA3* expression during the initial phases of trophectoderm specification remains an interesting question that deserves more detailed investigation in the future.

Positive feedback regulation of *TCF7* and *LEF1* following experimental WNT activation in primed hPSCs is reminiscent of some other tissues, for instance, in the context of colorectal cancer (Li et al., 2006; Mayer et al., 2020; Roose et al., 1999). Both Tcf7 and Lef1 contribute to paraxial mesoderm development, somite formation and limb development (Galceran et al., 1999), with mutants phenocopying Wnt3a-null mice (Takada et al., 1994). Although LEF1 has been described previously to be induced by active WNT/β-catenin signalling using either CHIR or recombinant WNT3A protein (Funa et al., 2015; Martyn et al., 2019), we also find *TCF7* is an additional uncharacterised target gene. The perdurance of TCF7 expression in definitive endoderm (albeit at low levels) and no LEF1 in our model suggests a temporal window of induction that might also be lineage specific (i.e. TCF7 and LEF1 expression might be maintained in mesoderm subtypes, especially in paraxial mesoderm, where WNT signalling is required to drive this specification; Mariani et al., 2021).

This analysis provides a basis for future experiments to investigate mechanisms of gene regulation of these important WNT-mediating TCF/LEF genes during early human development, comparing it to mouse. This analysis also provides a basis for formulating novel hypotheses about regulation of downstream gene expression by WNT signalling and any possible differences between TCF7-, LEF1-, TCF7L1- or TCF7L2-mediated downstream gene expression, and how the described differences in TCF/LEF deployment between human and the mouse paradigm might account for differences in subsequent gene expression (Martello et al., 2012). This analysis focused on expression of the four TCF/LEF genes; however, TCF7, LEF1 and TCF7L2 are known to express different mRNA and protein isoforms (Torres-Aguila et al., 2022), which might fine-tune the transcriptional response to WNT/β-catenin signalling in early human development.

### Limitations of the study

Our current TCF7 and LEF1 antibodies do not detect endogenously expressed dominant-negative isoforms, which might account for differences between mRNA and protein levels observed in this study. CHIR is a potent pharmacological inhibitor of GSK3 activity (Wu et al., 2015) and a widely used experimental tool to potently drive β-catenin into the nucleus, and this might not recapitulate a physiologically relevant mechanism. Moreover, we report more pronounced effects of CHIR (increasing *BRA, TCF7* and *LEF1* expression), in comparison to recombinant WNT3A protein, when human primed cells were treated. Currently, it remains unknown how the human embryo regulates endogenous WNT ligand secretion and how long is required to induce differentiation. Indeed, treating hPSCs with BMP4 induces *WNT3A* expression and thus stimulates WNT/β-catenin signalling (Martyn et al., 2019). Finally, whilst the focus of this study was on WNT/β-catenin-dependent signalling, future research should include studying the functions of β-catenin-independent (non-canonical) WNT signalling mechanisms involved in early human development.

## MATERIALS AND METHODS

### Human embryo thawing

Human embryo experiments at King's College London were performed under licence from the UK Human Fertilisation and Embryology Authority (research licence number: R0075) and independently reviewed by the UK National Health Service Research Ethics Committee (Reference: 06/Q0702/90). The patients who donated surplus embryos were provided with the necessary information about the research project and an opportunity to receive counselling. The informed consent included approval of the publication of the results in scientific journals. No financial inducements were offered for donations. Human embryo experiments were conducted in accordance with the principles expressed in the Declaration of Helsinki. Slow-frozen day 5 human blastocysts were thawed using a Blast thaw kit according to the manufacturer's instructions (Origio; 10542010A) and placed into drops of pre-equilibrated global medium (LifeGlobal; LGGG-20) supplemented with 5 mg/ml protein supplement (LifeGlobal; LGPS-605) and overlaid with mineral oil (Origio; ART-4008-5P). Embryos were cultured for 24 h and fixed at day 6 post-fertilisation in 4% paraformaldehyde for 1 h on ice.

### Human and mouse stem cell lines

The HNES1, HNES3 (Guo et al., 2016) and cR-H9 (Guo et al., 2017) cell lines were a generous gift from Professor Austin Smith (Living Systems Institute, University of Exeter, UK) after granted regulatory approval by the UK Stem Cell Committee. WA07 and WA09 were obtained from WiCell (Madison, WI, USA). IMR-90 and KOLF2.1J human induced pluripotent stem cell lines were a kind gift from Dr Eunchai Kang (Institute of Medical Sciences, University of Aberdeen, UK). E14Tg2a (Doetschman et al., 1987) and wild-type AinV18 ES cells were a kind gift from Todd Evans (Weill Cornell Medical College, New York, NY, USA) (Turbendian et al., 2013), and the JNST wild-type cell line (JNST #32) was derived in-house previously (Kraunsoe et al., 2023). Cell lines were all expanded in culture conditions that reflect their states of pluripotency and were regularly tested for mycoplasma contamination in-house. Cell lines regularly tested negative. DNA content analysis was performed to monitor the karyotypic status of all cell lines.

### Mouse embryonic stem cell culture

JNST #32, E14Tg2a and AinV18 (wild-type) mESCs were cultured on 0.15% gelatin (Gibco)-coated plates in 2i+LIF to maintain a state of naïve pluripotency, which consisted of N2B27 (Mulas et al., 2019) supplemented with 1 µM PD0325901 (Tocris), 3 µM CHIR99021 (Tocris) and 10 ng/ml mouse recombinant LIF (Cambridge Stem Cell Institute). Cells were passaged using Accutase (BioLegend) every 3–4 days. Epiblast stem cells (EpiSCs) were cultured to maintain primed pluripotency on 10 µg/ml bovine plasma fibronectin (Sigma Aldrich)-coated plates in AFX medium which consisted of N2B27 supplemented with 10 ng/ml human/mouse/rat recombinant Activin A (Peprotech), 12 ng/ml FGF2 (Cambridge Stem Cell Institute) and 2 µM XAV939 (Tocris). EpiSCs were passaged using Accutase every 2–3 days depending on growth rate and initial seeding density. 10 µM ROCKi (Y-27635, Tocris) was added to AFX medium for 24 h at passaging.

### Human naïve pluripotent stem cell culture

HNES1, HNES3 and cR-H9 cell lines were routinely maintained on gamma-irradiated DR4 mouse embryonic fibroblasts (iMEFs; Applied BioSystems) in N2B27 supplemented with 1 µM PD0325901, 2 µM XAV939 (Tocris), 2 µM Gö6983 (Tocris) and 10 ng/ml human recombinant LIF (Peprotech), termed PXGL as previously described (Bredenkamp et al., 2019; Guo et al., 2017). HNES cells were passaged every 3–4 days during exponential growth using Accutase for 12 min at 37°C and plated in PXGL with 10 µM ROCKi for 24 h. To improve attachment rate, Geltrex (Gibco) was diluted 1:4 with ice-cold DMEM (Gibco) and 30 µl was added per well of a six-well plate. Cells used for experiments were cultured for no more than five consecutive times. For t2iLiGö experiments, PXGL cells were passaged and the medium was switched to 1 µM PD0325901, 1 µM CHIR99021, 2 µM Gö6983 and 10 ng/ml LIF (Takashima et al., 2014).

For feeder-free culture, stock Geltrex solution was prediluted 1:4 with ice-cold DMEM and gently inverted. For coating plates, 1 ml of ice-cold DMEM was added to each well of a six-well plate with 50 µl of prediluted Geltrex solution. Plates were incubated overnight at 37°C.

### Capacitation of human naïve ES cells

HNES1 and HNES3 cells were capacitated as previously described (Rostovskaya et al., 2019). Briefly, HNES1 and HNES3 cells were plated at 40,000 cells per well of a six-well plate in PXGL+Y (PXGL containing 10 µM ROCKi) for 24 h. The next day, cells were washed once with DPBS (Gibco) and switched to N2B27 supplemented with 2 µM XAV939 (Tocris) for 10 days. After which, cells were passaged using Accutase (Biolegend) onto diluted Geltrex (1:800) in DMEM and AFX or StemFlex (Gibco) medium for at least three passages before being used for experimentation.

### Primed/conventional human pluripotent stem cell culture

Capacitated HNES1, HNES3 and conventional primed H9 ES cell lines were routinely cultured on Geltrex-coated plates (1:100 dilution overnight at 37°C) in StemFlex medium. Confluent cells were propagated using Accutase for 5 min at 37°C and replated in StemFlex medium supplemented with 10 µM ROCKi for 24 h. Similar results could also be obtained using AFX medium (see mouse embryonic stem cell culture)

### Hypoblast differentiation

Differentiation of HNES cells towards hypoblast cells was performed as previously described (Dattani et al., 2024). HNES cells were seeded on Geltrex-coated plates in PXGL with 10 µM ROCKi for 24 h (6 wells, 125,000 cells per well and individual Ibidi wells, 20,000 cells). The next day, the cells were washed twice with 1× PBS solution and the medium was exchanged to N2B27 supplemented with PDA83 (for HNES1, 1 µM PD0325901 and 1.5 µM A8301 (Stem Cell Technologies); for HNES3 and cR-H9, 1 µM PD0325901 and 1 µM A8301) for 24 h. The medium was then exchanged to N2B27 supplemented with 50 ng/ml human recombinant FGF4 (Thermo), 1.5 µM A8301 and 1 µM XAV939 for a further 48 h until the end of the assay.

### Trophectoderm differentiation

HNES cells were induced to differentiate into trophoblast-like cells as previously described (Guo et al., 2021). Naïve ES cells on day 3 or 4 (depending on growth) were passaged into PXGL medium with 10 µM ROCKi for 24 h. Cells were seeded at 125,000 cells per well of a six-well plate or 20,000 per individual Ibidi well. The next day, cells were washed once with DPBS and switched to differentiation medium that consisted of N2B27 supplemented with 1.5 µM PD0352901 and 1.5 µM A8301. The differentiation medium was refreshed daily and monitored for hallmark signs of differentiation (widespread epithelialisation after 24 h) for 72 h.

### nEnd differentiation

HNES cells were differentiated into naïve endoderm (nEND) cells as previously described (Linneberg-Agerholm et al., 2019; Mackinlay et al., 2021). To reduce cell death under feeder-free conditions (Geltrex-coated plates), the concentration of Activin A was reduced to 50 ng/ml. HNES cells were seeded on Geltrex-coated plates in PXGL with 10 µM ROCKi for 24 h at a density of 100,000 cells per well of a six-well plate and 20,000 cells per individual Ibidi well. The next day, the cells were washed twice with 1× PBS and exchanged to N2B27 supplemented with 50 ng/ml Activin A, 3 µM CHIR99021 and 10 ng/ml human LIF for 5–6 days. Medium was refreshed daily until the end of the assay.

### Generation of blastoids

Blastoids were generated using previously published protocols (Kagawa et al., 2022; Yanagida et al., 2021). Briefly, HNES1 and HNES3 cells were expanded until day 4 then dissociated using Accutase and counted. The cell number was adjusted to 100 cells per individual microwell of a 24-well Aggrewell plate in N2B27 for 24 h. The next day, the medium was gently changed to PALLY (Kangawa et al., 2022) for a further 48 h before being switched finally to A8301 and LPA (Tocris). After 96 h of induction, blastoids were collected and fixed for immunofluorescence microscopy.

### Amnion differentiation

Primed human ES cells were differentiated towards amnion cells as previously described (Guo et al., 2021; Rostovskaya et al., 2022). Cells were dissociated using Accutase and seeded onto Geltrex-coated plates at a seeding density of 75,000 cells per well of a six-well plate and 12,500 cells per individual Ibidi well (HNES1 cells, 100,000 cells per well of a six-well plate and 15,000 cells per Ibidi well) for 24 h in StemFlex with 10 µM ROCKi. The next day, cells were changed to StemFlex medium only for an additional 24 h. 48 h following seeding, cells were washed once with PBS solution then the medium was switched to N2B27 supplemented with 1 µM PD0325901, 1 µM A8301 and 50 ng/ml human recombinant BMP4 protein (Peprotech) for 72 h. The medium was refreshed daily until the end of the assay.

### Definitive endoderm differentiation

Human primed ES cell lines were differentiated into definitive endoderm in a stepwise manner using a previously published protocol with minor modifications (Loh et al., 2014). Cells were dissociated using Accutase and passaged onto Geltrex-coated plates (75,000 cells per well of a six-well plate and 15,000 per individual Ibidi well) in StemFlex medium supplemented with 10 µM ROCKi. The next day, cells were exchanged to StemFlex medium only for an additional 24 h. After 48 h, cells were washed once with PBS solution and their medium was switched to DE1 (N2B27 with 100 ng/ml Activin A, 20 ng/ml FGF2, 5 ng/ml BMP4 and 3 µM CHIR99021). PI-103 was omitted. After 24 h, the medium was changed to DE2 [N2B27 with 100 ng/ml Activin A, 10 ng/ml FGF2 and 300 ng/ml human recombinant NOGGIN protein (Peprotech)] for an additional 48 h until the end of the assay. All media were refreshed daily and extra was added from 48–72 h to accommodate larger cell numbers.

### Immunofluorescence of cell culture samples

Ibidi-treated 8-well chamber slides (Ibidi) were used for immunofluorescence microscopy. Cells were fixed with 4% paraformaldehyde solution for 20 min at room temperature (RT) and subsequently washed three times with 1× PBS. Samples that were not processed immediately were stored at 4°C in PBS. Samples were permeabilised using 0.5% Triton X-100 in PBS for 25 min at RT then blocked [5% donkey serum (Sigma-Aldrich), 5% BSA (Sigma-Aldrich) with 0.25% Triton X-100 in PBS] for 1 h at RT on an orbital shaking platform. Primary antibodies were diluted accordingly in blocking solution and incubated overnight at 4°C. The next day, samples were washed three times with PBST (PBS with 0.02% Tween-20) and incubated with secondary antibodies and DAPI (Invitrogen, 1:5000 dilution) in blocking solution for 2 h at RT on an orbital shaking platform set at 65 r.p.m. Samples were washed three times with 1× PBST and, finally, changed to PBS prior to imaging. Details of antibodies are shown in Table S1.

### Immunofluorescence of human blastocysts

Immunofluorescence staining was performed as described previously (Balestrini et al., 2024). Briefly, embryos were washed three times in 1×PBS with 0.1% Tween-20. Embryos were permeabilised with 1× PBS with 0.5% Triton X-100 and then blocked in blocking solution (10% donkey serum in 1× PBS with 0.1% Triton X-100) for 1 h at RT. Embryos were incubated with primary antibodies diluted in blocking solution overnight at 4°C on rotating shaker. Embryos were washed for 20 min and then incubated with secondary antibodies diluted in 1× PBS with 0.1% Triton X-100 for 1 h at RT. Embryos were washed in 1× PBS with 0.1% Triton X-100 for 20 min. Finally, embryos were placed in 1× PBS with 0.1% Triton X-100 with Vectashield and DAPI mounting medium (Vector Lab; H-1200; 1:30 dilution) in μ-Slide 8-well coverslip dishes for confocal imaging (Ibidi, 80826). Imaging was performed using a Nikon AXR inverted confocal microscope with 40× objective. Details of antibodies are shown in Table S1.

### Confocal microscopy

All images of all cell lines were acquired using either a Leica SP5 or LSM-880 confocal microscope with 20× objectives, using either Leica, Nikon Elements or ZEN Black software. To minimise crosstalk between the channels, laser intensities were set and not altered throughout each imaging session. Imaging emission spectra tracks were reduced to limit bleed-through. To normalise or improve the signal–noise ratio for each fluorophore, the digital gain was adjusted accordingly. TCF/LEF expression was normalised to human and mouse primed samples, and TCF7/LEF1 was cross-examined with human primed ES cells treated with CHIR for 24 h. These image settings were saved and used for each experiment and were not altered. Signal to noise reduction was performed to decrease the non-specific binding of the Alexa Fluor 488 anti-mouse IgG secondary antibody to unspecified components of Geltrex, which was used to coat the Ibidi imaging chambers.

### Intracellular flow cytometry

Cells were dissociated using Accutase, resuspended in ice-cold 1% paraformaldehyde and fixed for 1 h at 4°C. Samples were washed once with 1× PBS and permeabilised with 70% chilled methanol in PBS for 1 h on ice. Blocking was performed using 5% donkey serum, 5% BSA in PBS for 1 h at 4°C. Primary antibodies (1:100 dilution) were added directly to blocking buffer and incubated for 1 h. Samples were washed three times with 1:5 diluted blocking buffer. Secondary antibody staining (1:1000 dilution) was performed for 1 h at 4°C, and samples were subsequently washed three times. Finally, samples were resuspended into 300 μl of wash buffer and stored at 4°C for analysis (BD Fortessa). Data analysis was carried out using Flow Jo software. For each experiment, total cells were gated using SSC-A and FSC-A, and subsequent single cells were identified using a rectangle gate with FSC-H and FSC-A. Unstained and secondary-antibody-only controls were used to establish positive and negative gated single cells. At least 20,000 single gated events were captured. Details of antibodies are shown in Table S1.

### RNA extraction, cDNA synthesis and qRT-PCR

Samples were lysed in six-well plates using lysis buffer (Qiagen), and RNA was extracted using a Qiagen RNeasy column prep kit using the manufacturer's instructions (Qiagen). RNA was eluted into RNA storage solution (Thermo) and was evaluated for integrity by gel electrophoresis. 500 ng of RNA was reverse transcribed with SuperScript IV (Invitrogen) as per the manufacturer's instructions; however, only 0.5 µl of SuperScript IV reverse transcriptase was used per reaction. cDNA was then diluted 1:10 with nuclease-free water. Reverse transcription-qPCR (qRT-PCR) was performed using Power Up SyBR Green MasterMix (Applied Biosystems) in 384-well white qPCR plates (Applied Biosystems) as technical quadruplicates (5 µl reactions). Threshold cycle (cT) values were measured using the LightCycler II machine. cT values were obtained using the delta delta cT method (Livak and Schmittgen, 2001), and the average of *UBC* and *ACTB* was used as an internal reference for normalisation of relative expression. Expression data were analysed using GraphPad Prism V10 along with statistical analysis. Unless specified, all experiments were performed with at least three independent cell lines (HNES1, HNES3 and cR-H9). Primer pair sequences are shown in Table S2.

### Statistics and reproducibility

All statistical analyses were carried out using GraphPad Prism V10. Individual details of statistical methods used for each experiment are presented in each figure legend. Immunofluorescence microscopy experiments were performed in at least three independent cell lines: HNES1, HNES3 (Guo et al., 2016) and cR-H9 (Guo et al., 2017), and images presented were representative of five independent fields of view. qRT-PCR experiments were performed on all three independent cell lines with technical quadruplicates. Flow cytometry experiments were performed on all three independent cell lines, and at least 20,000 events were recorded. Once optimised, all cell lines behaved in a similar manner and yielded consistent and reproducible results in our hands. TCF/LEF antibodies purchased from Santa Cruz (see Table S1 in red) exhibited varying efficacy, extensive lot-to-lot variability and required high concentrations to obtain data. The authors advise caution when using these antibodies. Alternatively sourced TCF/LEF antibodies used in this study (green, Table S1) are presented in each figure panel and were comprehensively validated across multiple cell samples and species (human, mouse and rat).

## Supplementary Material

10.1242/joces.264257_sup1 Supplementary information
