# Supplementary material for: WNT-mediating TCF/LEF transcription factor gene expression in early human pluripotency and cell lineages differs from the rodent paradigm
Source: J Cell Sci. 2025 Sep 26;138(18):jcs264257. doi: 10.1242/jcs.264257 (PMC12516193; doi:10.1242/jcs.264257)
Supplement: Supplementary information [file joces-138-264257-s1.pdf]

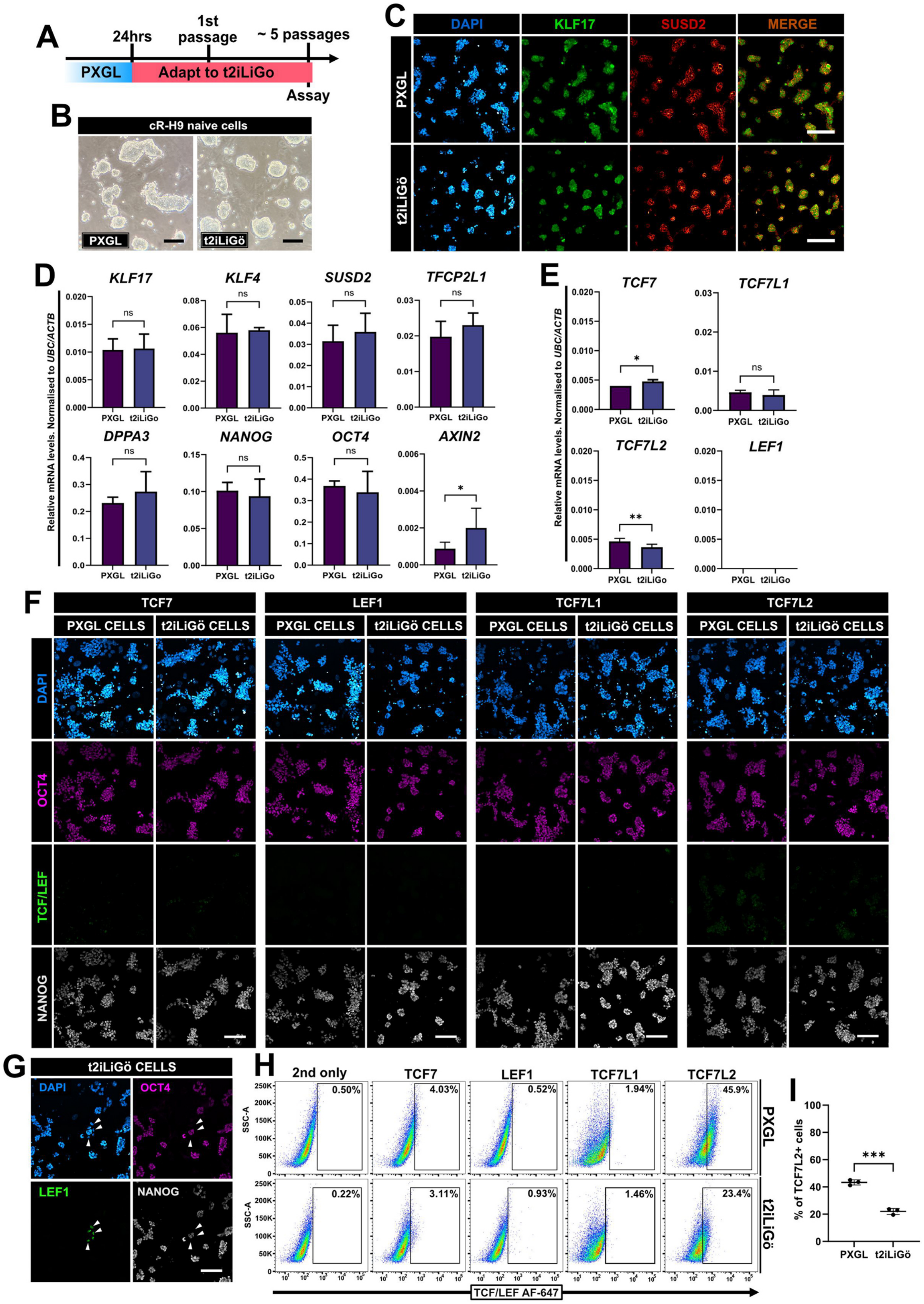

Journal of Cell Science • Supplementary information

Fig. S1. TCF/LEF expression in PXGL vs t2iLiGö naïve human pluripotent stem cells

(A) Diagrammatic outline to adapt HNES1, HNES3 and cR-H9 PXGL cells to t2iLiGö culture conditions over 5 passages prior to experiments.

(B) Phase contrast images of cR-H9 naïve ES cells cultured in PXGL or t2iLiGö after 96 hours.

(C) Immunofluorescence of cR-H9 naïve ES cells culture in either PXGL or t2iLiGö conditions for KLF17 (green) and SUDS2 (red). Nuclei were counterstained with DAPI. Scale bars = 100 µm.

(D) RT-qPCR analysis of naïve (*KLF17, KLF14, SUSD2, TFCP2L1* and *DPPA3*) and core (*NANOG*, *OCT4*) pluripotency marker and *AXIN2* (readout for active WNT signalling). Relative mRNA levels were normalised to *UBC* and *ACTB*. Student's paired t-test (* = $p< 0.05$ and ns = not significant). Error bars indicate ±SD, N=3.

(E) RT-qPCR assay of human naïve pluripotent stem cells cultured in either PXGL or t2iLiGö conditions for TCF/LEF gene expression. Relative mRNA levels were normalised to *UBC* and *ACTB*. Student's paired t-test (* = $p<0.05$, ** = $p< 0.01$, and ns = not significant). Error bars indicate ±SD, N=3.

(F) Immunofluorescence of cR-H9 naïve cells cultured in either PXGL or t2iLiGö for OCT4 (magenta), TCF7/LEF1/TCF7L1/TCF7L2 (green) and NANOG (grey). Nuclei were counterstained with DAPI. Scale bars = 100 µm.

(G) Immunofluorescence of cR-H9 naïve ES cells cultured in t2iLiGö for 5 passages at 72 hours since passaging for OCT4 (magenta), LEF1 (green) and NANOG (grey).

(H) Flow cytometry plots of TCF/LEF expression in either PXGL or t2iLiGö culture conditions.

(I) Quantification of $TCF7L2^{+}$ naïve cells in PXGL and t2iLiGö conditions. Student's paired t-test (*** = $p<0.001$ and ns = not significant). Error bars indicate ±SD, N=3.

Journal of Cell Science • Supplementary information

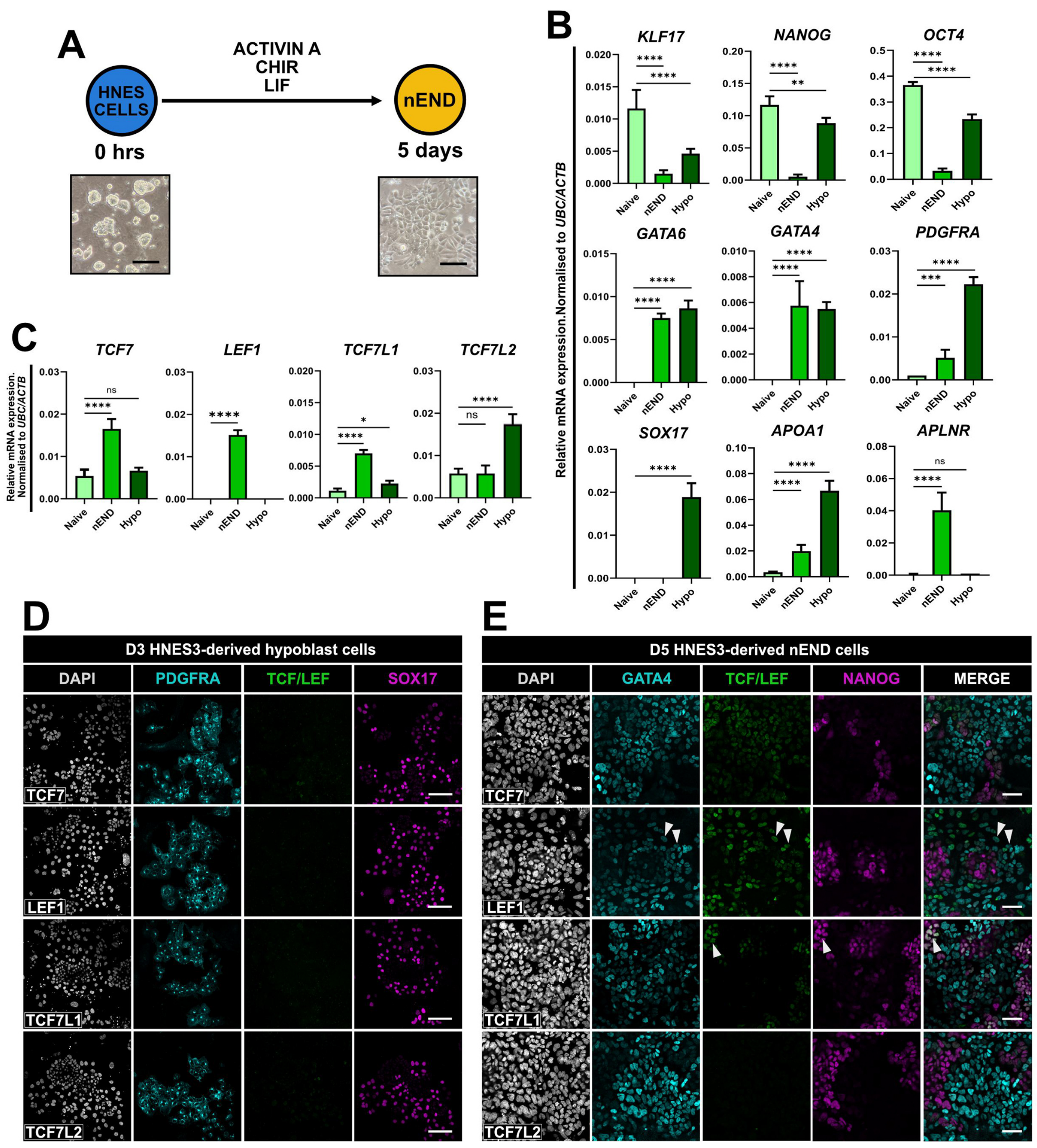

Fig. S2. Comparison of TCF/LEF factor expression in nEND and hypoblast cells

(A) Experimental schematic for the differentiation of HNES cells using ACTIVIN, CHIR and LIF (ACL) for 5 days to generate nEND cells. Scale bars = 100 μm.

(B) qRT-PCR of naïve, nEND and hypoblast cells for pluripotency and hypoblast markers. Relative mRNA levels were normalised to *UBC* and *ACTB*. Student's paired t-test (** = *p*< 0.01, **** = *p*<0.0001 and ns = not significant). Error bars indicate ±SD, N=3.

(C) RT-qPCR of naïve, nEND and hypoblast cells for all four TCF/LEF genes. Relative mRNA levels were normalised to *UBC* and *ACTB*. Student's paired t-test (* = $p< 0.05$, **** = $p<0.0001$ and ns = not significant). Error bars indicate ±SD, N=3.

(D) Immunofluorescence of HNES3-derived hypoblast cells for PDGFRA (cyan), TCF/LEF factors (green) and SOX17 (magenta). Scale bars = 100 µm.

(E) Immunofluorescence of HNES3-derived nEND cells for GATA4 (cyan), TCF/LEF factors (green) and NANOG (magenta). Arrows for LEF1 indicating LEF1/GATA4 co-expressing cells and arrow for TCF7L1 indicate TCF7L1/NANOG co-expressing cells. Scale bars = 100 µm.

Journal of Cell Science • Supplementary information

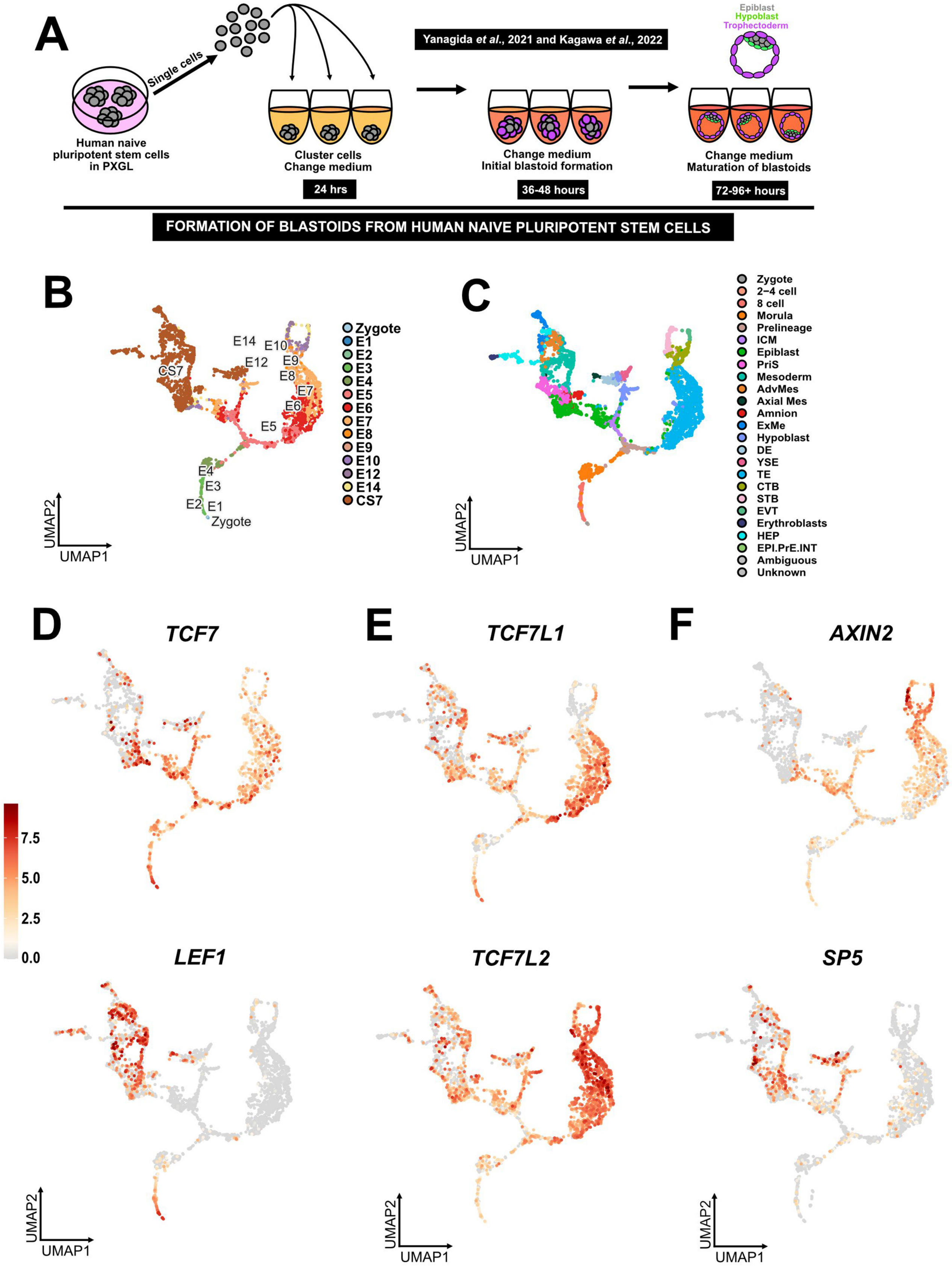
A
Single cells
Yanagida et al., 2021 and Kagawa et al., 2022
Epiblast
Hypoblast
Trophectoderm
Human naive pluripotent stem cells in PXGL
Cluster cells
Change medium
24 hrs
Change medium
Initial blastoid formation
36-48 hours
Change medium
Maturation of blastoids
72-96+ hours
FORMATION OF BLASTOIDS FROM HUMAN NAIVE PLURIPOTENT STEM CELLS
B
Zygote
E1
E2
E3
E4
E5
E6
E7
E8
E9
E10
E12
E14
CS7
UMAP1
UMAP2
C
Zygote
2−4 cell
8 cell
Morula
Prelineage
ICM
Epiblast
PriS
Mesoderm
AdvMes
Axial Mes
Amnion
ExMe
Hypoblast
DE
YSE
TE
CTB
STB
EVT
Erythroblasts
HEP
EPI.PrE.INT
Ambiguous
Unknown
D
TCF7
LEF1
E
TCF7L1
TCF7L2
F
AXIN2
SP5
7.5
5.0
2.5
0.0

Fig. S3. Blastoid formation and RNA-seq analysis of TCF/LEF expression in early human embryogenesis

(A) Protocol diagram to generate blastoids from human naïve pluripotent stem cells routinely cultured in PXGL medium.

(B) UMAP plot of integrated human embryo datasets based on developmental stage (embryonic day) from zygote to Carnegie stage 7 (CS7, gastrula).

(C) UMAP plot of annotated linages from human embryo datasets.

(D) UMAP plots for *TCF7* and *LEF1* expression.

(E) UMAP plots for *TCF7L1* and *TCF7L2* expression.

(F) UMAP plots of WNT target genes *AXIN2* and *SP5*.

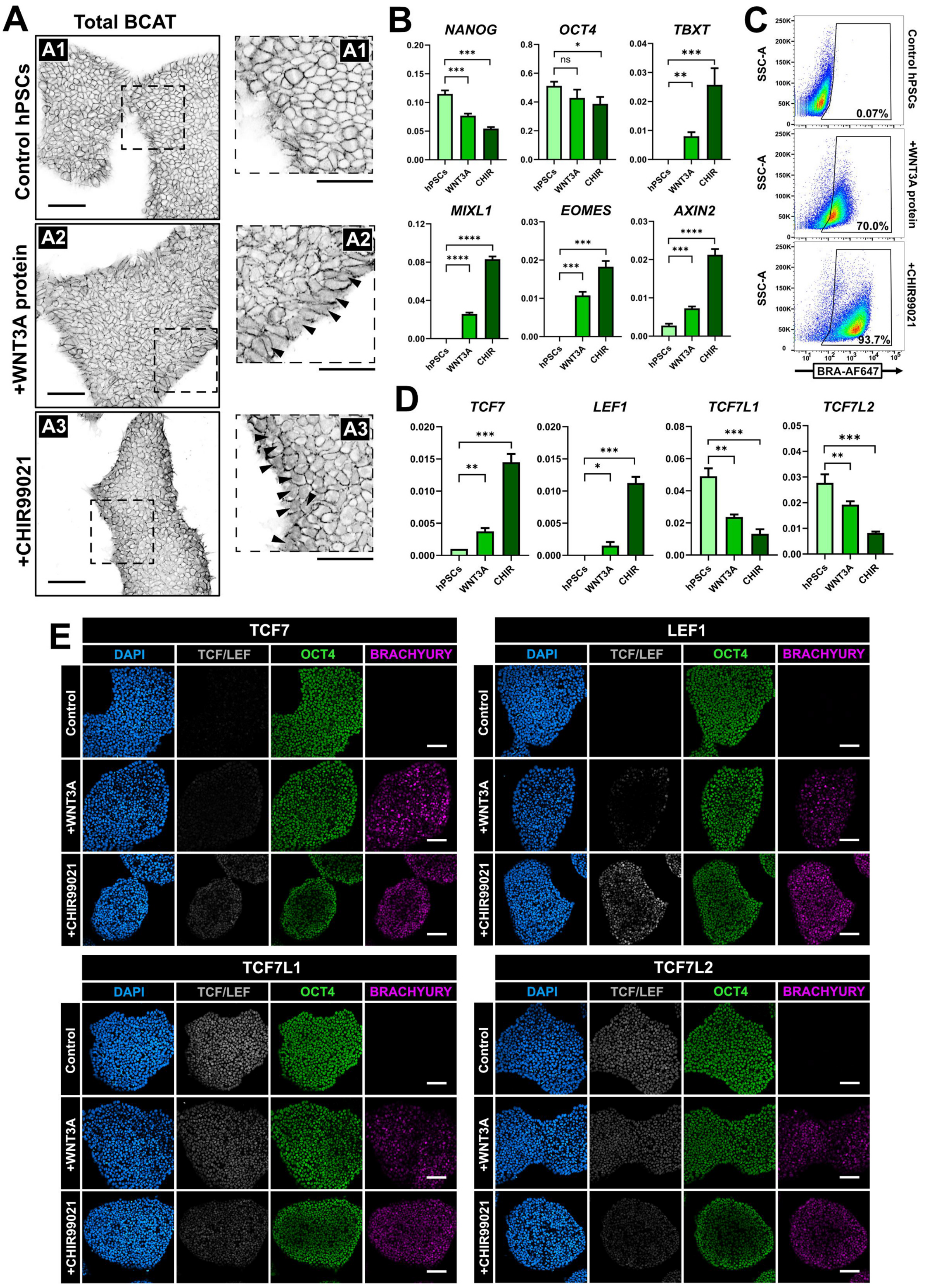

Journal of Cell Science • Supplementary information

Fig. S4. TCF7 and LEF1 are upregulated in primed hPSCs in response to WNT activation with either CHIR or recombinant WNT protein

(A) Immunofluorescence of control or treated pHNES3 cells (with 300 ng/mL mouse recombinant WNT3A protein or 3 µM CHIR99021) for 24 hours. Scale bars = 100 µm. Inserts representative for each culture condition. Scale bars = 200 µm. N=3.

(B) RT-qPCR of pluripotency and gastrulation markers after 24 hours in control and treated hPSCs. Relative mRNA levels were normalised to *UBC* and *ACTB*. Student's paired t-test (* = p<0.05, ** = p< 0.01, *** = p<0.001, **** = p<0.0001 and ns = not significant). Error bars indicate ±SD, N=3.

(C) Flow cytometry plots for BRACHYURY^{+} cells in control hPSC or following a 24-hour treatment with either recombinant 300 ng/mL WNT3A protein or 3 µM CHIR. N=3.

(D) RT-qPCR of all four TCF/LEF transcription factor genes in control hPSCs vs treated for 24 hours. Relative mRNA levels were normalised to *UBC* and *ACTB*. Student's paired t-test (* = p<0.05, ** = p< 0.01, *** = p<0.001). Error bars indicate ±SD, N=3.

(E) Representative immunofluorescence of pHNES3 cells treated with either recombinant WNT3A or CHIR for 24 hours for TCF7/LEF1/TCF7L1/TCF7L2 (grey), OCT4 (green) and BRACHYURY (magenta). Scale bars = 100 µm. N=3.

Journal of Cell Science • Supplementary information

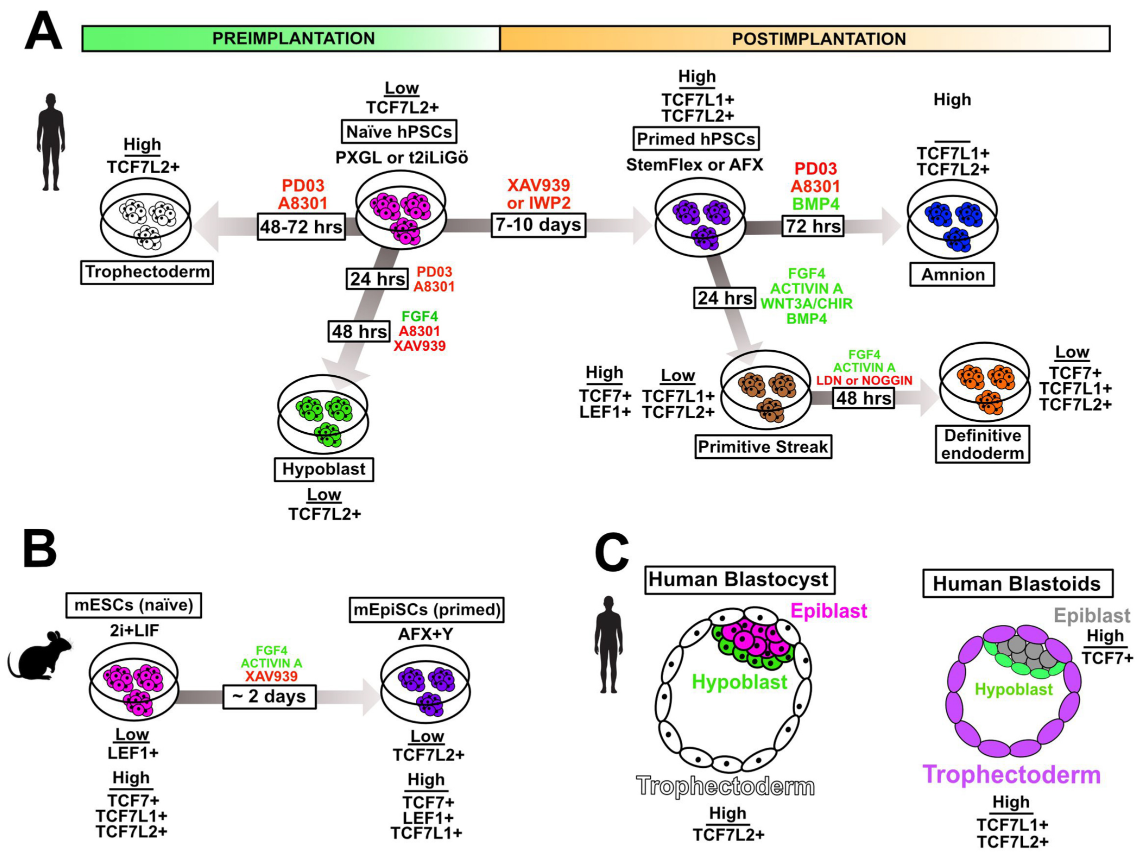

D

Cells and Tissues:	Mouse	Human			Mouse	Human				Human blastoids (3D stem cell models)	Preimplantation human blastocysts
	Naïve PSCs		Extra-embryonic		Primed PSCs		Extra-embryonic	Embryonic lineages			
	mESC	nPSCs	Hypo	TE	mEpiSCs	hPSC	Amnion	Pr. Streak	Endoderm		
Genes: *TCF7*	High				High			High	Low	Epi (High)	
LEF1	Low*				High			High			
TCF7L1	High				High	High	High	Low	Low	TE (High)	
TCF7L2	High	Low	Low	High	Low	High	High	Low	Low	TE (High)	TE (High)

PSCs: Pluripotent stem cells; **Hypo:** Hypoblast; **TE:** Trophectoderm; **pr. streak:** primitive streak, **Epi:** Epiblast. * signifies heterogeneous expression

Fig. S5. A roadmap of TCF/LEF transcription factor expression throughout early human development

(A) Diagrammatic representation of human development modelled exclusively using pluripotent stem cells featuring signalling conditions, gene markers and TCF/LEF factor expression.

(B) Proposed TCF/LEF factor expression in mouse ESCs (mESCs, naïve state) and EpiSCs (mEpiSCs, primed state).

(C) Proposed TCF/LEF factor expression in human preimplantation blastocysts and blastoid model.

(D) Table summary of TCF/LEF factors expressed in different cell and embryo samples from this study.

Journal of Cell Science • Supplementary information

Table S1. Antibodies used in this study

Target	Vendor	Item code	Dilutions
**OCT4**	Santa Cruz	SC-5279	1:200 (IF)
**NANOG**	R&D	AF1997	1:200 (IF)
**KLF17**	Atlas Antibodies	HPA024629	1:200 (IF)
**KLF4**	CST	4038S	1:100 (IF)
**TFCP2L1**	R&D	AF5726	1:50 (IF)
**GATA3**	eBioScience	14-9966-80	1:200 (IF) 1:100 (FC)
**GATA3**	Abcam	Ab1399428	1:200 (IF)
**PDGFRA**	Abcam	Ab203491	1:100 (IF)
**BRACHYURY**	R&D	AF2085	1:200 (IF)
**GATA4**	eBioScience	14-9980-82	1:100 (IF)
**SOX17**	R&D	AF1924	1:200 (IF)
**TCF7**	Santa Cruz	SC-271453	1:100 (IF)
**TCF7L1**	Santa Cruz	SC-166411	1:100 (IF)
**TCF7L2**	Santa Cruz	SC166699	1:100 (IF)
**LEF1**	Santa Cruz	SC-374412	1:100 (IF)
**TCF7**	**Invitrogen**	**MA5-14965**	**1:200 (IF) 1:100 (FC)**
**TCF7L1**	**PGT labs**	**14519-1-AP**	**1:200 (IF) 1:100 (FC)**
**TCF7L2**	**CST**	**2569S**	**1:200 (IF) 1:100 (FC)**
**LEF1**	**CST**	**2230S**	**1:200 (IF) 1:100 (FC)**
**β-Catenin**	Abcam	Ab16051	1:200 (IF)
**Anti-mouse 488**	Invitrogen	A-21202	1:1000 (IF and FC)
**Anti-rat 488**	Invitrogen	A-21208	1:1000 (IF and FC)
**Anti-rabbit 555**	Invitrogen	A-31572	1:1000 (IF and FC)
**Anti-rabbit 647**	Invitrogen	A-31573	1:1000 (IF and FC)
**Anti-goat 647**	Invitrogen	A-21447	1:1000 (IF and FC)

Journal of Cell Science • Supplementary information

Table S2. qRT-PCR primer pairs

Gene	Forward sequence (5'-3')	Reverse sequence (5'-3')
UBC	CACCTGGTGCTCCGTCTCAG	TCGATGGTGTCACTGGGCTC
ACTB	GTGGATCAGCAAGCAGGAGT	GCAACTAAGTCATAGTCCGC
GAPDH	GACATCAAGAAGGTGGTGAAGC	GTCCACCACCCTGTTGCTGTAG
KLF17	GCTGCCCAGGATAACGAGAAC	ATCTCTGCGCTGTGAGGAAAG
KLF4	ACAGTCTGTTATGCACTGTGGTTTCA	CATTTGTTCTGCTTAAGGCATACTTGG
TFCP2L1	CGTTTAAGCAGAACGAGAATGGG	TTTCATAGGACGGCTGGTATTTC
DPPA3	AGACCAACAAACAAGGAGCCT	CCCATCCATTAGACACGCAGA
NANOG	GCAGAAGGCCTCAGCACCT	AGGTTCCCAGTCGGGTTCA
OCT4	CGAAAGAGAAAGCGAACCAG	AACCACACTCGGACCACATC
GATA6	GAGGCTTGCTGAAAGAGTGAGAGAAGA	CCTAGTCCTGGCTTCTGGAAGTG
GATA4	GGAAGCCCAAGAACCTGAAT	GTTGCTGGAGTTGCTGGAA
SOX17	GACCGCACGGAATTTGAACA	GGACACCACCGAGGAAATGG
PDGFRA	GGGCACGCTCTTTACTCCAT	GCTGGCAGAGGATTAGGCTC
APOA1	CCCAGTTGTCAAGGAGCTTT	TGGATGTGCTCAAAGACAGC
GATA3	CTCATTAAGCCCAAGCGAAGG	GGTTGTGGTGGTCTGACAGTTC
GATA2	AGCAAGGCTCGTTCCTGTTC	CACAGGCATTGCACAGGTAGT
TFAP2A	GTTACCCTGCTCACATCACTAG	TCTTGTCACTTGCTCATTGGG
VGLL1	TGCCTCCCGGCTCAGTTCACT	CCCAGTGGTTTGGTGGTGTA
VTCN1	TCTGGGCATCCCAAGTTGAC	TCCGCCTTTTGATCTCCGATT
IGFBP5	ACCTGAGATGAGACAGGAGTC	GTAGAATCCTTTGCGGTCACAA
TBXT	TGCTTCCCTGAGACCCAGTT	GATCACTTCTTTCCTTTGCATCAAG
MIXL1	GGTACCCCGACATCCACTTG	TAATCTCCGGCCTAGCCAAA
EOMES	CAACATAAACGGACTCAATCCCA	ACCACCTCTACGAACACATTGT
APLNR	GGTGCAGTGCTACATGGACT	ATGGTGAAGGGCACCACAAA
CXCR4	CACCGCATCTGGAGAACCA	GCCCATTTCCTCGGTGTAGTT
FGF17	CCAACTCTACAGCAGGAC	CATACAGATGTACTTCTCACTC
CER1	TTCTCAGGGGGTCATCTTGC	ATGAACAGACCCGCATTTCC
AXIN2	TATCCAGTGATGCGCTGACG	CGGTGGGTTCTCGGGAAATG
TCF7	TGATGCTAGGTTCTGGTGTACC	CTTGTGTCTTCAGGTTGCG
TCF7L1	ATGTCCAGCCTGGTCTCC	GATTTCACGCTCACTGCAG
TCF7L2	AAGTGCCAGTGGTGCAGC	GGATATATCTGGAGGGTGCGG
LEF1	CATATGATTCCCGGTCCTC	CTGAGGCTTCACGTGCAT

Journal of Cell Science • Supplementary information
